# Multiaxial validation of a finite element model of the intervertebral disc with multigenerational fibers to establish residual strain

**DOI:** 10.1002/jsp2.1145

**Published:** 2021-03-21

**Authors:** Harrah R. Newman, John F. DeLucca, John M. Peloquin, Edward J. Vresilovic, Dawn M. Elliott

**Affiliations:** ^1^ Department of Biomedical Engineering University of Delaware Newark Delaware USA; ^2^ Department of Orthopaedic Surgery University of Pennsylvania Medical Center Hershey Pennsylvania USA

**Keywords:** annulus, finite element model, intervertebral disc, residual, strain, stress

## Abstract

Finite element models of the intervertebral disc are used to address research questions that cannot be tested through typical experimentation. A disc model requires complex geometry and tissue properties to be accurately defined to mimic the physiological disc. The physiological disc possesses residual strain in the annulus fibrosus (AF) due to osmotic swelling and due to inherently pre‐strained fibers. We developed a disc model with residual contributions due to swelling‐only, and a multigeneration model with residual contributions due to both swelling and AF fiber pre‐strain and validated it against organ‐scale uniaxial, quasi‐static and multiaxial, dynamic mechanical tests. In addition, we demonstrated the models' ability to mimic the opening angle observed following radial incision of bovine discs. Both models were validated against organ‐scale experimental data. While the swelling only model responses were within the experimental 95% confidence interval, the multigeneration model offered outcomes closer to the experimental mean and had a bovine model opening angle within one SD of the experimental mean. The better outcomes for the multigeneration model, which allowed for the inclusion of inherently pre‐strained fibers in AF, is likely due to its uniform fiber contribution throughout the AF. We conclude that the residual contribution of pre‐strained fibers in the AF should be included to best simulate the physiological disc and its behaviors.

## Graphical abstract

We validated a disc model with residual contributions due to swelling‐only and a multigeneration model with residual contributions due to both swelling and AF fiber pre‐strain against organ‐scale uniaxial, quasi‐static and multiaxial, dynamic mechanical tests as well as tested the models’ ability to mimic the opening angle observed following radial incision of bovine discs. The swelling only model was within the confidence interval, though the multigeneration model was closer to the experimental mean; the better outcomes for the multigeneration model is likely due to its uniform fiber contribution throughout the AF. We conclude that the residual contribution of pre‐strained fibers in the AF should be included to best simulate the physiological disc and its behaviors.

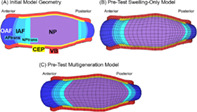

## 1 INTRODUCTION

Finite element models of the intervertebral disc aim to incorporate its complex geometry and material properties to predict the disc's multiaxial mechanics and address research questions that cannot be tested through experimentation. The constituent relations used in a model of the disc should describe the nonlinear, anisotropic, osmotic and biphasic properties in order to effectively simulate the mechanical behavior of the physiological disc.^1^, ^2^, ^3^, ^4^ Our lab previously developed a disc model with such properties and validated it against uniaxial, quasi‐static compression tests.^4^ In our model, disc constituent material properties were obtained from individually testing the disc's tissue components. The model as a whole was then validated with organ‐scale testing. The model replicated mechanical outcomes in uniaxial quasi‐static slow ramp, creep, and stress relaxation tests very well^4^; however, when subsequently applied to dynamic, multiaxial tests, the model was unable to match the nonlinear experimental responses (Figure S1).

The disc is comprised of an inner nucleus pulposus (NP) and outer layers of annulus fibrosus (AF). The NP is rich in proteoglycans that enable it to maintain a high water content; this fluid is essential to the NP pressurization which enables the disc to withstand high compressive loads.^5^, ^6^, ^7^, ^8^, ^9^ The AF also has high proteoglycan content to support compression, but importantly, it consists of fibrous, concentric layers with an angle‐ply fiber structure which enable the AF to withstand circumferential tension.^5^, ^6^, ^10^, ^11^, ^12^ To the superior and inferior of the NP and AF are porous cartilage endplates (CEP) which control fluid and nutrient exchange between the disc and vertebral bodies (VB).^8^, ^13^, ^14^, ^15^, ^16^ The combination of these components enables the disc to withstand large, multi‐axial, dynamic loading during regular daily activities.

**FIGURE 1:**
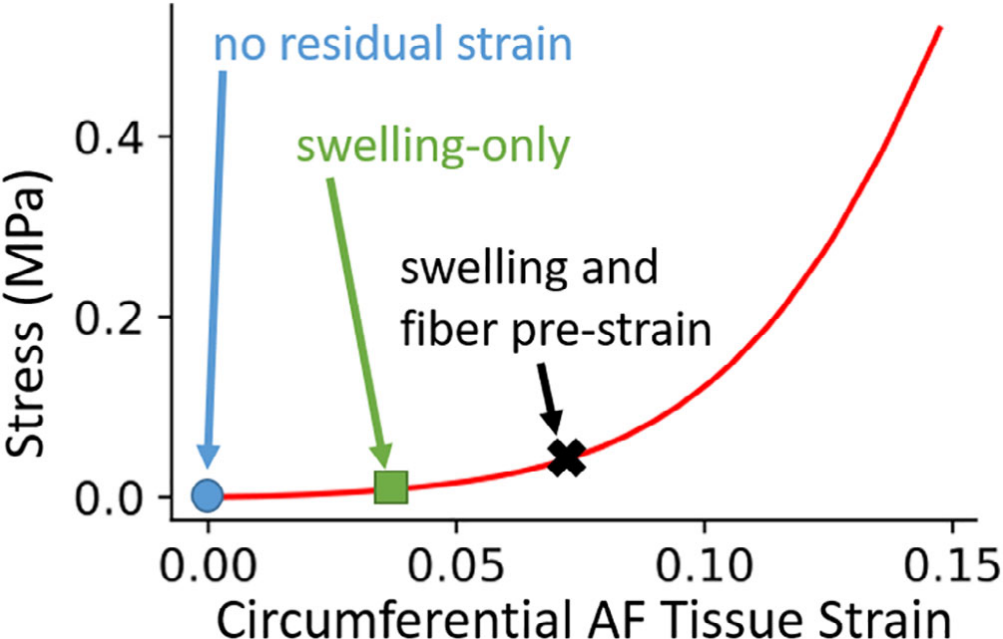
AF tissue testing to determine material properties (red stress–strain response) is performed on excised tissue with residual strain released (blue circle); however, the intact disc is subject to significant pre‐strain. The disc finite element model is initiated in a stress‐ and strain‐free reference configuration (blue circle) and the AF pre‐strain can be increased by imposing residual strain due to swelling (green square) or due to both swelling and fiber pre‐strain (black X); with the overarching goal of matching the disc state at the condition of interest (e.g., the start of motion segment testing, as in the tests utilized for validation herein)

Significant residual strain is present in the AF, but its inclusion in finite element models is inconsistent and potentially insufficient.^17^, ^18^ Bovine discs removed from the endplates and then cut radially, opened up as the internal residual strain is released.^18^ These outcomes demonstrate that the disc's residual strain is partially released in the absence of swelling pressure and further by the relaxation and recoil of the AF fibers. Therefore, the disc possesses two types of residual strain, 1) due to swelling, and 2) due to inherently pre‐strained fibers in the AF. The high proteoglycan content in the disc sets up a fixed charge density gradient that draws and holds fluid in the disc space enabling a buildup of swelling pressure. The fibers present in the AF develop an inherent pre‐strain as a result of growth and development.^17^, ^19^, ^20^ The experiments conducted on physiological discs have mechanical contribution from both of these residual strains whereas model simulations do not. The material properties used in models are acquired from tissue tests which require the extraction of individual disc components. Tissue extraction releases residual strains and leaves their contribution unaccounted for in constituent testing and in the resultant material properties used in finite element models (Figure 1).^4^, ^21^ While many models have included residual strain contributions due to swelling,^1^, ^2^, ^4^, ^22^, ^23^, ^24^, ^25^, ^26^, ^27^, ^28^, ^29^ none to date have explicitly included residual contribution from pre‐strained AF fibers. We hypothesize our previous model lacked sufficient residual strain which inhibited its performance in dynamic axial compression and torsion testing scenarios (Figure S1).

We sought to update our previous model and develop a process to establish pre‐strained AF fibers in the model. In order to incorporate pre‐strained fibers we utilized the multigeneration feature in FEBio, which allows for constitutive relationships of the model to be established at different reference configurations.^30^

The first objective of this study was to incorporate residual strain due to swelling and multigeneration fibers in a finite element model of human disc and validate it against uniaxial quasi‐static and multi‐axial dynamic tests. We compared a swelling‐only model, with AF residual strain caused by swelling, and a multigeneration model, with AF residual strain caused by both swelling and inherent AF fiber pre‐strain. A parametric analysis was conducted for the multigeneration model with several parameter combinations to investigate the impact of fiber‐induced residual strain, and to determine the values best suited for simulating experimental outcomes and by extension, the physiological disc.

The second objective of this study was to simulate the bovine disc incision experiment by Michalek and coauthors to demonstrate the model's residual strain.^18^ The AF opening gap following radial incision is a measure of residual strain. We simulated the radial incision experiment using both the swelling‐only and multigeneration models, calculated the AF opening gap, and compared it against the previously published experimental outcomes.^18^

## 2 METHODS

We expanded upon our previous work to establish a swelling‐only model and developed a novel multigeneration model which featured residual strain in the AF due to both swelling and pre‐strained fiber contributions. For both models, it is important to note that material properties were based on tissue‐level experiments and were not tuned to fit organ‐level experimental outcomes (Table 1).^4^, ^5^, ^6^, ^13^, ^21^, ^31^, ^32^, ^33^, ^34^ For the parametric study of the multigeneration model, we varied two parameters, axial displacement (∆*H*) and twist angle (Ω), as the disc height and torsional rotation impacted the fiber pre‐strain imposed and will be described in greater detail later in Section 2.3.2. Bovine and human discs possess similar water and proteoglycan content, and their mechanical responses scale with disc size; therefore, the same constitutive equations and material properties for the human AF and NP were also utilized for the bovine model and only geometric differences were included.^35^, ^36^, ^37^, ^38^

**TABLE 1:** Material properties for outer annulus fibrosus (OAF), inner annulus fibrosus (IAF), nucleus pulposus (NP), and cartilage endplates (CEP)

	Matrix properties (Cortes+ 2012, Cortes+ 2014, DeLucca+ 2016)						Fiber properties (Elliott+ 2001, O'Connell+ 2009, Jacobs+ 2013, Cassidy+ 1989)			
	Modulus *E*_*m*_ (MPa)	Poisson ratio *ν*	Exponential stiffening coefficient *β*_*m*_	Hydraulic permeability *k*_0_ (mm^4^/Ns)	Exponential strain dependence *M*	Fixed charge density (mM)	Modulus *E*_*f*_ (MPa)	Transition stretch ratio *λ*_o_	Fiber angle *θ* (± degrees)	Toe region power law exponent *β*_*f*_
OAF	0.018	0.24	3.4	0.0047	5.75	44	15.6	1.028	31.0	4
AFtrans	0.023	0.20	2.8	0.0036	4.60	50	10.3	1.025	38.5	4
IAF	0.026	0.16	2.1	0.0025	3.50	55	6.9	1.023	41.5	4
NPtrans	0.045	0.20	1.5	0.0016	2.71	217	3.0	1.020	44.5	4
NP	0.065	0.24	0.95	0.00056	3.79	379	N/A	N/A	N/A	N/A
CEP	0.305	0.18	0.29	0.00056	3.79	248	N/A	N/A	N/A	N/A

*Note*: AFtrans is a 1‐element wide transitional layer between the OAF and IAF; similarly, NPtrans is a 1‐element wide transitional layer between the IAF and the NP. The material properties are from tissue‐level experimental work and were either calculated or fit via inverse finite element modeling.

### 2.1 Initial geometry and mesh for human disc model

**FIGURE 2:**
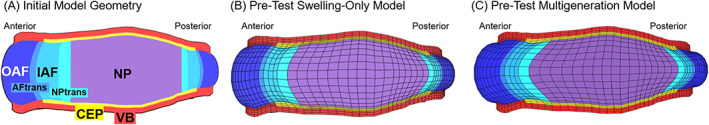
A, Sagittal view of the initial model geometry for with disc constituent labels for the outer annulus fibrosus (OAF), annulus fibrosus transitional layer (AFtrans), inner annulus fibrosus (IAF), nucleus pulposus transitional layer (NPtrans), nucleus pulposus (NP), cartilage endplates (CEP) and vertebral bodies (VB). B, Swelling‐only model after swelling at fixed height (*H* = 11 mm), immediately prior to preload and test protocol. C, Multigeneration model after swelling and fiber deposition at prescribed height (*H* = 11.5 mm), immediately prior to preload and test protocol

The three‐dimensional geometry of the model was created from the mean shape of seven human L4/L5 discs of Pfirrmann degeneration grade 3.^4^, ^39^ A custom Matlab script meshed the mean disc to 10,625 hexahedral elements and subsequent swelling conditions established the initial bulged shape. The disc model includes distinct constitutive models and material properties for the NP, AF, CEP, and VB (Figure 2). The properties of the AF vary radially, therefore it was modeled as having an outer layer (OAF) and inner layer (IAF) with a transitional single element layer between them (AFtrans). Similarly, a single element transitional layer was created between the IAF and the NP (NPtrans) (Figure 2; Table 1).

### 2.2 Constitutive models and material properties

#### 2.2.1 Matrix

The AF, NP, and CEP all included extrafibrillar matrix with a Holmes‐Mow constitutive equation.^40^ Holmes‐Mow matrix is an isotropic, hyperelastic material model which is widely used to model disc.^3^, ^4^, ^21^, ^29^, ^41^ The strain‐energy function for the Holmes Mow matrix is:

(1) $$W\left(I_{1},I_{2},J\right)=\frac{\lambda +2\mu }{4\beta_{m}}\left(e^{\frac{\beta_{m}}{\lambda +2\mu }\left(\left(2\mu -\lambda \right)\left(I_{1}-3\right)+\lambda \left(I_{2}-3\right)-\left(\lambda +2\mu \right)\ln\left(J^{2}\right)\right)}-1\right)$$

where *I*_1_ and *I*_2_ are the right Cauchy‐Green tensor invariants, *J* is the Jacobian of the deformation gradient, *β*_*m*_ is the matrix stiffening coefficient, and *λ* and *μ* are the Lamé parameters which can be related to the Young's modulus *E*_*m*_ and Poisson's ratio *ν* as follows:

(2) $$\lambda =\frac{E_{m}}{\left(1+\nu \right)\left(1-2\nu \right)}\;\text{and}\;\mu =\frac{E_{m}}{2\left(1+\nu \right)}$$

*E*_*m*_, *ν*, and *β*_*m*_ were specified for each constituent based on tissue‐level compression tests (Table 1).^6^, ^21^

#### 2.2.2 Permeability and Donnan swelling

Holmes‐Mow permeability was utilized to achieve isotropic, strain‐dependent permeability according to the following permeability tensor:^40^, ^42^

(3) $$k=\left[k_{o}{\left(\frac{J-\varphi_{0}}{1-\varphi_{0}}\right)}^{2}e^{\frac{M}{2}\left(J^{2}-1\right)}\right]I$$

where *k*_o_ is the isotropic hydraulic permeability in the reference state and *M* is the exponential strain‐dependence coefficient, both of which are specified for each disc constituent based on tissue tests (Table 1).^6^, ^13^, ^21^

The fluid content is established through Donnan equilibrium swelling pressure which established the pressure that would be produced if the matrix were to be populated with charged ions and surrounded by an external bath solution that contained counter‐ions. The Donnan equilibrium response imposes the Cauchy stress for the material as:

(4) $$\sigma_{\mathrm{Don}}=-\mathrm{\pi I}$$

where *π* is the osmotic pressure, a function of the gas constant (*R*), temperature (*T*), osmotic coefficient (*Φ*), fixed‐charge density (*c* ^*F*^) with reference to the initial fixed‐charge density ($c_{0}^{F}$), and the osmolarity of the external bath (${\bar{c}}^{*}$):

(5) $$\pi =\mathrm{RT\Phi}((\sqrt{{\left(c^{F}\right)}^{2}+{\left({\bar{c}}^{*}\right)}^{2}})-{\bar{c}}^{*})$$

where the instantaneous fixed‐charge density depends on the reference configuration as follows:

(6) $$c^{F}=\left(\frac{\varphi_{0}^{w}}{J-1+\varphi_{0}^{w}}\right)c_{0}^{F}$$

The bath osmolarity was 300 mOsm/L and the reference state fixed charge density $c_{0}^{F}$ is specified for each constiuent based on tissue‐level experiments (Table 1).^6^

#### 2.2.3 Fibers

The AF constitutive model included fibers within the isotropic matrix to provide tensile stiffness. The nonlinear fibers were described by a strain energy density:^43^, ^44^

(7) $$\Psi_{n}\left(I_{n}\right)=\left\{\begin{array}{c} 0\;I_{n}<1\\ \frac{E_{f}}{4\beta_{f}\left(\beta_{f}-1\right)}{\left(I_{0}-1\right)}^{2-\beta_{f}}{\left(I_{n}-1\right)}^{\beta_{f}}\;1\leq I_{n}\leq I_{0}\\ E_{f}\left(I_{0}^{1/2}-I_{n}^{1/2}\right)+\frac{E_{f}}{2}\left(I_{n}-I_{0}\right)\left(I_{0}\left(\frac{1}{2\left(\beta_{f}-1\right)}+1\right)-\frac{1}{2\left(\beta_{f}-1\right)}\right)+\Psi_{0}\;I_{0}<I_{n} \end{array}\right\}$$

where the initial stretch parameter was defined as $I_{0}=\lambda_{0}^{2}$; similarly, the instantaneous stretch was $I_{n}=\lambda_{n}^{2}$, and the initial strain energy density was $\Psi_{0}=\frac{E_{f}}{4\beta_{f}\left(\beta_{f}-1\right)}{\left(I_{0}-1\right)}^{2}$.

The fiber modulus (*E*_*f*_), toe‐region power law exponential (*β*_*f*_), and transition stretch ratio (*λ*_*o*_) for each AF region, were specified based on tensile tissue testing of the AF (Table 1)^31^, ^32^, ^34^ and the fiber angles were based on optical microscope image analysis.^5^ Each AF element had two fiber sets, oriented at the positive and negative fiber angle (*θ*). The NP and CEP did not include fiber contributions.

#### 2.2.4 Rigid bodies and boundary conditions

The VB were assigned a Neo‐Hookean constitutive relation with Young's modulus 10,000 MPa and Poisson's ratio of 0.3.^4^, ^45^, ^46^ The superior and inferior surfaces of the model were defined by rigid bodies. The inferior rigid body was fixed in all degrees of freedom for the entirety of all testing scenarios. The superior rigid body was used to impose all loads, displacements and rotations as specified for each testing protocol (Table 2).

**TABLE 2:** Test case specific protocols that followed after the pre‐test conditions shown in Figure 3

Test case	Test type	Preload	Test control	Test condition	Experimental data source
Slow ramp	Quasi‐Static Uniaxial	Load at Δ*H*	Axial load (z)	1 N/s to 2000 N	O'Connell+ 2011
Creep	Quasi‐static uniaxial	Load at Δ*H*	Axial load (z)	200 N/s to 1000 N, then hold	O'Connell+ 2011
Stress relaxation	Quasi‐static uniaxial	50 N preload	Axial displacement (z)	5% compression in 5 seconds, then hold	Yoder+ 2014 Showalter+ 2016
Axial compression	Dynamic multiaxial	270 N preload	Axial load (z)	18 N/s to 900 N	DeLucca+ 2019
Torsion	Dynamic multiaxial	270 N preload	Axial Rotation (Rz)	0.08°/s to 3°	DeLucca+ 2019
Bending	Dynamic multiaxial	270 N preload	Sagittal Rotation (Ry)	0.08°/s to 3°	DeLucca+ 2019
Flexion	Dynamic multiaxial	270 N preload	Coronal Rotation (Rx)	0.08°/s to 3°	DeLucca+ 2019

*Note*: Preload was enacted in axial force control with all other degrees of freedom fixed, the preload was held for 12 hours in accordance with the experimental protocols. For the test sequence, the test control was the only degree of freedom specified, all other degrees of freedom were fixed.

### 2.3 Human disc model protocol

The swelling‐only model did not include pre‐strained fibers, such that all residual strain was exclusively swelling‐induced. The swelling‐only protocol involved three stages: swelling at fixed height (*H* = 11 mm) (Methods 2.3.1, Figure 2B), axial preload (Methods 2.3.3, Table 2, Figure 7A‐D), and loading simulation for test case of interest (Methods 2.3.3, Table 2, Figures 8A‐D, 9A‐E).

**FIGURE 3:**
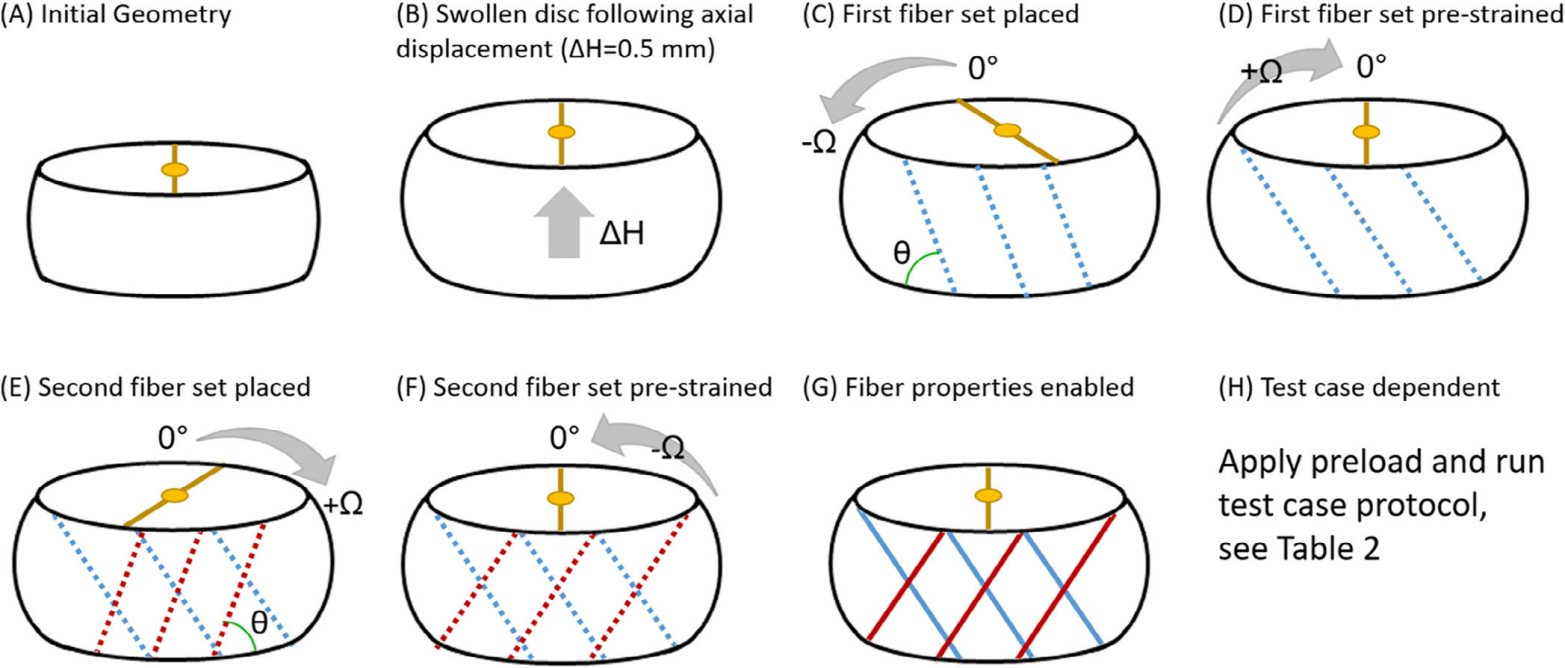
Protocol schematic showing (A) the initial, unswollen state with superior reference marker (yellow). B, Axial displacement (Δ*H* = 0.5 mm) was applied and the disc was swollen to Donnan equilibrium. C, The disc was twisted −Ω from the neutral position and the first fiber set was deposited with negligible fiber modulus, then (D) it was returned to neutral position, which stretched the first fiber set. E, The disc was then twisted +Ω, and the second fiber set was deposited and then (F) it was then returned to the neutral position so that both fiber sets were stretched. G, Once both fiber sets were placed and stretched, the material properties of the fibers were increased to full value (see Table 1). This process established a disc model with residual stress contributions from both swelling and multigenerational AF fibers. H, The applied preload and test case protocols that followed are outlined in Table 2

The multigeneration model included residual strain contributions from both swelling and AF fiber pre‐strain. The fiber pre‐strain was dependent upon the disc geometry at the time of fiber placement. Two parameters: axial displacement (∆*H*) and torsional twist angle (Ω) were used to evaluate the impact of varying fiber pre‐strain on organ‐scale outcomes. We found a disc height of 11.5 mm (∆*H*=0.5 mm) was necessary to place fibers that produced reasonable uniaxial outcomes in slow ramp and creep (Figures S2 and S3) and this was therefore used in all subsequent multigeneration models herein. The twist angle was varied to Ω = 2° , 3° , 4° to investigate how torsional placement of fibers impacted model outcomes. To this end, the multigenerational protocol involved four stages: swelling with axial displacement (∆*H* = 0.5) (Methods 2.3.1, Figures 2C and 3B), multigenerational fiber deposition (Methods 2.3.2, Figure 3C‐G), axial preload (Methods 2.3.3, Table 2, Figure 7E‐H), and loading simulation for test case of interest (Methods 2.3.3, Table 2, Figures 8E‐H and 9F‐J).

#### 2.3.1 Swelling

All models were initiated with a bulged AF (Figure 2A) and the fixed charge density in all tissues was zero. The fixed charge densities were then increased to full value (Table 1), altering the disc's stress state and resulting in a swollen disc geometry. The swelling‐only model possessed both fiber sets at model initiation, time zero, while the multigeneration model did not have any fibers in the AF during initial swelling. For the swelling‐only model, the disc height was fixed at *H* = 11 mm. For the multigeneration model, the disc height was increased while the tissue swelled to *H* = 11.5 mm (see Figure S2 for rationale). All models were allowed to reach the appropriate height and achieve Donnan equilibrium (Figures 2B,C and 3B).

#### 2.3.2 Fiber implementation (multigeneration model only)

The swelling‐only model possessed both fiber sets at model initiation; the following fiber implementation process is relevant for only the multigeneration model.

The multigeneration approach was used to add pre‐strained AF fibers to establish fiber residual strain independent of swelling residual strain. The extra‐fibrillar matrix was established at time zero in a non‐stressed state while the fibers were deposited in a stressed reference configuration that differed from the neutral state of the model. This provided for the disc model to possess stretched fibers creating fiber‐induced, circumferential residual stress in the AF. The stressed reference configuration was achieved by twisting the disc model about the spinal axis. The twist (Ω) was varied between model runs to 2°, 3°, and 4°.

At the end of the swelling phase the disc was in a neutral position (Figure 3B), it was then twisted −Ω degrees to a deformed state (Figure 3C). While the disc was in the deformed state the first fiber set was positioned at +θ, according to angles specified in Table 1. The disc was then twisted back to the neutral position which stretched the first fiber set (Figure 3D). From the neutral position the disc was twisted +Ω degrees to the opposing deformed state and the second fiber set was positioned at −θ (Figure 3E). Then disc was returned to a neutral position, where both fiber sets were now stretched (Figure 3F). Once both fiber sets were placed and the disc was in a neutral position, the material properties of the fibers were applied at their full values (Table 1) while boundary conditions were held fixed (Figure 3G). This process ultimately resulted in the multigeneration model: a swollen disc model (*H* = 11.5 mm) with pre‐strained crisscross fiber sets in the AF (Figure 1B, Figure 3G). For comparison, the swelling‐only model did not undergo this process and was, therefore, a swollen disc model (*H* = 11 mm) with crisscross fiber sets in the AF.

#### 2.3.3 Test cases

Seven test cases were utilized to validate the models and select the optimal multigeneration model parameters, axial displacement (∆*H*), and twist angle (Ω). Our previous model^4^ was validated against uniaxial quasi‐static tests; slow ramp and creep tests mimicked the experimental protocol of Reference 47 and the stress relaxation test mimicked the protocol of Reference 48. These tests were repeated on the current models to ensure agreement and no loss in the current model's ability to simulate these outcomes.^4^, ^47^, ^48^ Furthermore, the present models were tested in multiaxial dynamic tests which mimicked the experimental protocol in Reference 49 for axial compression, torsion, bending, and flexion tests.

For the dynamic tests, the preload was calculated by the same process utilized in the experiment, where the cross‐sectional area of the disc was estimated by *A* = 0.84 * *L*_right/left_ * *L*_anterior/posterior_ and the preload necessary to achieve physiologically relevant nucleus pressurization was calculated as $\text{preload}=\frac{0.2\;\mathrm{MPa}*A}{1.5}$.^49^, ^50^ The disc length measurements (L) were taken from the images of the discs used to develop the model geometry (n = 7). The preload calculated for the dynamic tests was 270 N, this load was imposed and allowed to equilibrate in the simulation over a 12‐hour period, as was done in the experimental tests, prior to the test case.^49^ The test case loading/rotation imposed were those from the final experimental cycle of the slowest test frequency for each test condition (Table 2).^49^

### 2.4 Human disc model outcomes

#### 2.4.1 Nonlinear disc response

The average disc response and associated 95% confidence interval were calculated for all experimental test cases: slow ramp (n = 4), creep (n = 4), stress relaxation (n = 5), axial compression (n = 8), torsion (n = 8), bending (n = 8), and flexion (n = 8).^4^, ^47^, ^48^, ^49^ To quantify the model's ability to replicate experimental outcomes, a normalized mean square error (NMSE) was calculated as follows:

(8) $$\text{NMSE}=\frac{\sum_{x\left(0\right)}^{x\left(n\right)}{\left[Y{\left(x\right)}_{\text{model}}-Y{\left(x\right)}_{\exp\text{mean}}\right]}^{2}}{\sum_{x\left(0\right)}^{x\left(n\right)}{\left[Y{\left(x\right)}_{95\%\mathrm{CI}}-Y{\left(x\right)}_{\exp\text{mean}}\right]}^{2}}$$

where *Y*(*x*) is the parameter of interest, for example in slow ramp *Y*(*x*) = *Load*(*time*). The mean square error between the model and experimental mean was normalized by the mean square error of the 95% confidence interval and experimental mean. This normalization enables evaluation of the model's ability to match experimental outcomes across different protocols. The closer the NMSE is to zero, the better the fit, and an NMSE greater than one indicates that the model outcome is outside of the 95% confidence interval for that particular test case.

#### 2.4.2 Model geometry

**FIGURE 4:**
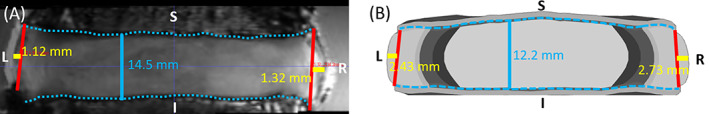
A, MRI of L4‐L5 disc following overnight 50 N load (Yoder 2014+, Showalter 2016+). Cyan dots on the superior/inferior edges were used to calculate the median disc height from a mid‐coronal MRI slice. Red reference lines connect the outermost points of the vertebral bodies. The lateral bulge was measured by the yellow line, from the end of the vertebral bodies reference (red line) to the outermost point of the disc. B, Mid‐coronal image of the multigeneration model after equilibration to 50 N load. The dashed cyan lines represent the superior/inferior disc surfaces which were used to calculate the median disc height. Disc bulge was calculated in the same manner as used for the MRI discs

In addition to checking the model's test case outcomes, we also sought to validate the model's geometry to ensure it effectively mimicked physiological discs. The L4/L5 discs that underwent MRI and were used to construct the initial model geometry were also scanned following an overnight 50 N load (n = 7) (Figure 4).^48^, ^51^ This load was imposed on the model following swelling and fiber placement (in the multigen model) such that the model's geometry could be compared to MRI data. The discs' height from MRI was calculated from a mid‐coronal slice, where points along the superior and inferior disc boundary were defined and the median distance between the superior and inferior markings was taken to be the disc height (Figure 4A). The height of the disc model was calculated as the median difference between the superior and inferior disc surfaces (Figure 4B). The left‐right bulge for both the MRI discs and disc model was determined by measuring the disc's lateral extrusion beyond the endplates. The bulge was measured from an endplate reference line to the outermost disc boundary (Figure 4A‐B).

#### 2.4.3 Fiber stress and strain

The stress and strain of the AF fibers was quantified on an element‐by‐element basis in the fiber direction. The coordinate axes were rotated to align with the fiber direction and the instantaneous deformation gradient with respect to the deformation gradient at the time of fiber deposition was calculated. This ultimately enabled calculation of the right Cauchy‐Green strain from which the strain along the fiber direction was determined. The fiber strain in conjunction with the fibers' defined material properties (Table 1) allowed for the calculation of fiber stress. For further details about the fiber stress and strain calculations please see supplemental section 6.5 Calculation of the Fiber Strain and Stress.

#### 2.4.4 Cauchy stress and Lagrange strain in disc

The stress and strain state of the multigeneration model at key time points was quantified in terms of local anatomic axes. Cauchy stress and Lagrange strain were transformed from *x*, *y*, *z* directions to the axial, radial, and circumferential directions in accordance with our prior work.^4^, ^39^, ^48^, ^51^ The directional transformation enabled improved interpretation of the stresses and strains with respect to the disc structure.

### 2.5 Bovine disc model protocol

In order to demonstrate our models' ability to mimic the observed bovine disc opening outcome,^18^ we simulated the bovine disc as a cylinder. The constitutive equations and material properties were the same as those used for the human disc model (Table 1). The model geometry was chosen such that the end‐of‐swelling diameter matched the experimentally measured mean bovine disc size.^52^ The swelling‐only model was initiated with fibers present in the AF, then allowed to swell to osmotic equilibrium as explained in Methods 2.3.1. The multigeneration model was swollen, then subjected to multigeneration fiber placement with a twist angle of 3°, according to the process previously explained in Methods 2.3.2. For both models, following swelling and fiber placement (multigeneration model only), the radial cut was simulated by release of planar boundary conditions along the radial interior surfaces on either side of the cut. The opening gap after the radial cut (W_i_) was quantified and compared with the experimental data.^18^ Additional experimental work was conducted with partial and full nucleus detachment, but we were unable to simulate these experiment with our current model as they were conducted in open air while our model assumes immersion in a bath solution.^18^, ^53^

## 3 RESULTS

In general, for the human disc, both the swelling‐only and multigeneration models performed well in comparison to multiaxial experimental data. The multigeneration model with twist angle Ω = 3° produced the best set of outcomes across all seven test cases. For the bovine disc, both the swelling‐only and multigeneration models had a positive opening angle; the multigeneration model opening was closer to that seen experimentally.^18^ All human disc models had a run‐time of less than an hour and the bovine disc model had a run‐time of less than 10 minutes.

### 3.1 Mechanical loading test cases for human disc model

**FIGURE 5:**
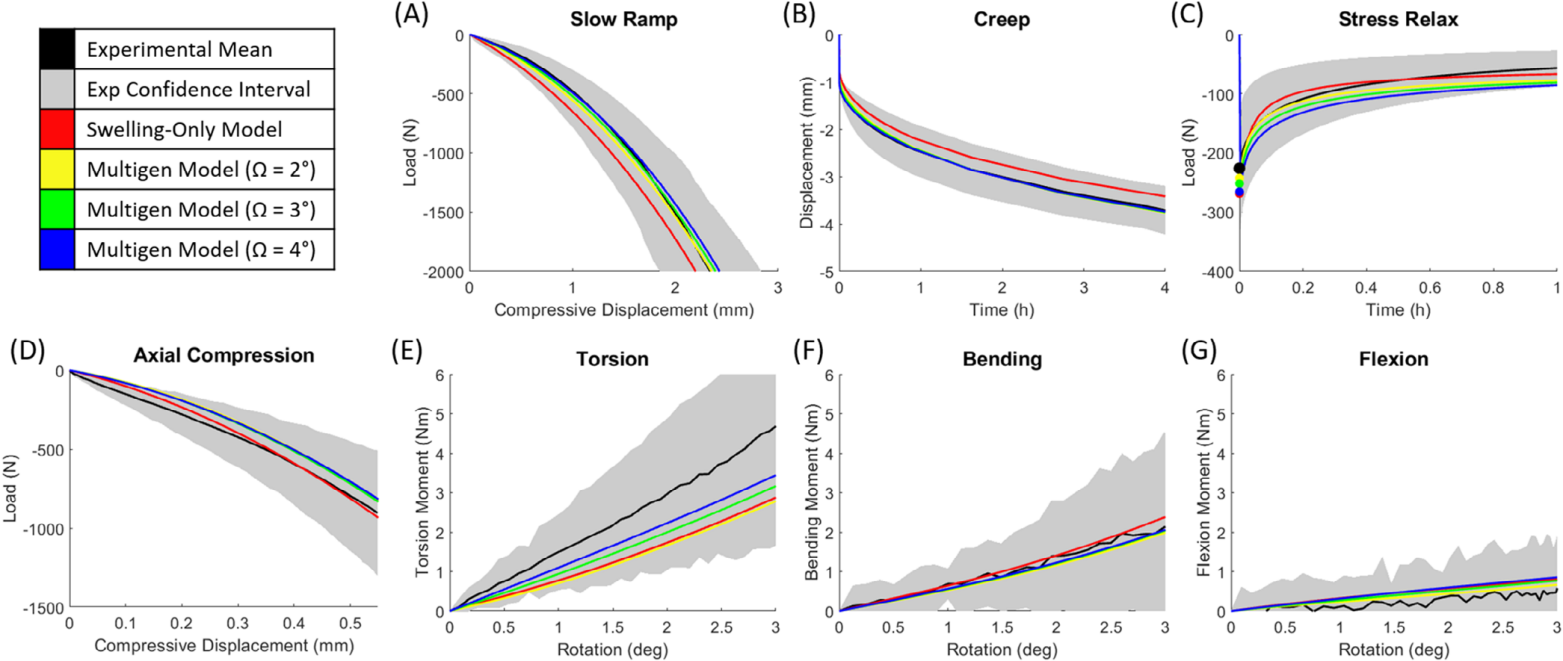
Experimental data ±95% confidence interval is shown with model outcomes for swelling‐only and multigeneration models with Δ*H* = 0.5 mm and variable twist angle (Ω). All models lie within the 95% confidence interval with for all tests. The multigeneration models perform better than swelling‐only in slow ramp (A), creep (B), and torsion (E). The swelling‐only model is better in stress relaxation (C) and axial compression (D). Increasing the twist angle in the multigeneration model (from Ω = 2° to 4°) improves the match to experimental data for torsion (E), but worsens the match for stress relaxation (C). All models sufficiently recapitulate bending (F) and flexion (G) outcomes

**FIGURE 6:**
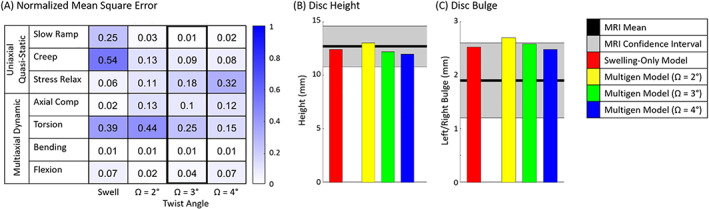
A, Normalized mean square error (NMSE) values are shown for the swelling‐only model and multigeneration model with varying twist angle (Ω). NMSE values closer to zero indicate a model response closer to the experimental mean. B and C, Disc height and bulge from the models equilibrated to a 50 N load compared to MRI data of discs under a 50 N load. B, All models were within expected height limits. C, All models are in the upper range of disc bulge

The nonlinear load‐displacement model response was compared to quasi‐static and viscoelastic experimental data and to dynamic multiaxial experimental data and the error was calculated. In a quasi‐static axial slow ramp, the multigeneration models performed better (NMSE < 0.05) than the swelling‐only model (NMES = 0.25) (Figures 5A and 6A). Similarly, in quasi‐static creep, the multigeneration models (NMSE < 0.15) performed better than the swelling‐only model (NMSE = 0.54) (Figures 5B and 6A). In contrast, in stress relaxation, the swelling‐only model (NMSE = 0.06) performed better than the multigeneration models (NMSE > 0.11) (Figures 5C and 6A). The multiaxial dynamic response of all models matched the experimental response in axial compression (NMSE < 0.15), bending (NMSE = 0.01), and flexion (NMES < 0.1) (Figures 5D‐G and 6A). The dynamic torsion response varied across multigeneration models (0.15 < NMSE < 0.44) and the swelling‐only model performed adequately (NMSE = 0.39) (Figures 5E and 6A).

Three versions of the multigeneration model were tested with fiber twist angles of Ω = 2°, 3°, and 4°, to evaluate the impact of the multigeneration model input of fiber twist angle on the model's mechanical outcomes. The variation in twist angle had minimal impact on slow ramp, creep, axial compression, bending, and flexion responses, where the maximum difference across twist angles was only ΔNMSE = 0.05. However, variation in twist angle had a significant impact on the stress relaxation response (ΔNMSE = 0.21) and torsion response (ΔNMSE = 0.29). The stress relaxation response improved with decreasing the twist angle; conversely, the torsion response improved with increasing twist angle. The multigeneration model with twist angle Ω = 3° was selected as the optimal twist angle, this decision was primarily driven by the necessary compromise between the stress relaxation and torsion responses.

### 3.2 Human disc model geometry

All models maintained a disc height within the experimental confidence interval after equilibration with a 50 N load (Figure 6B). The models produced lateral bulge in the upper range of that seen in MRI; for the multigeneration model, bulge reduced as twist angle increased (Figure 6C).

### 3.3 Model state: End of 270 N preload

**FIGURE 7:**
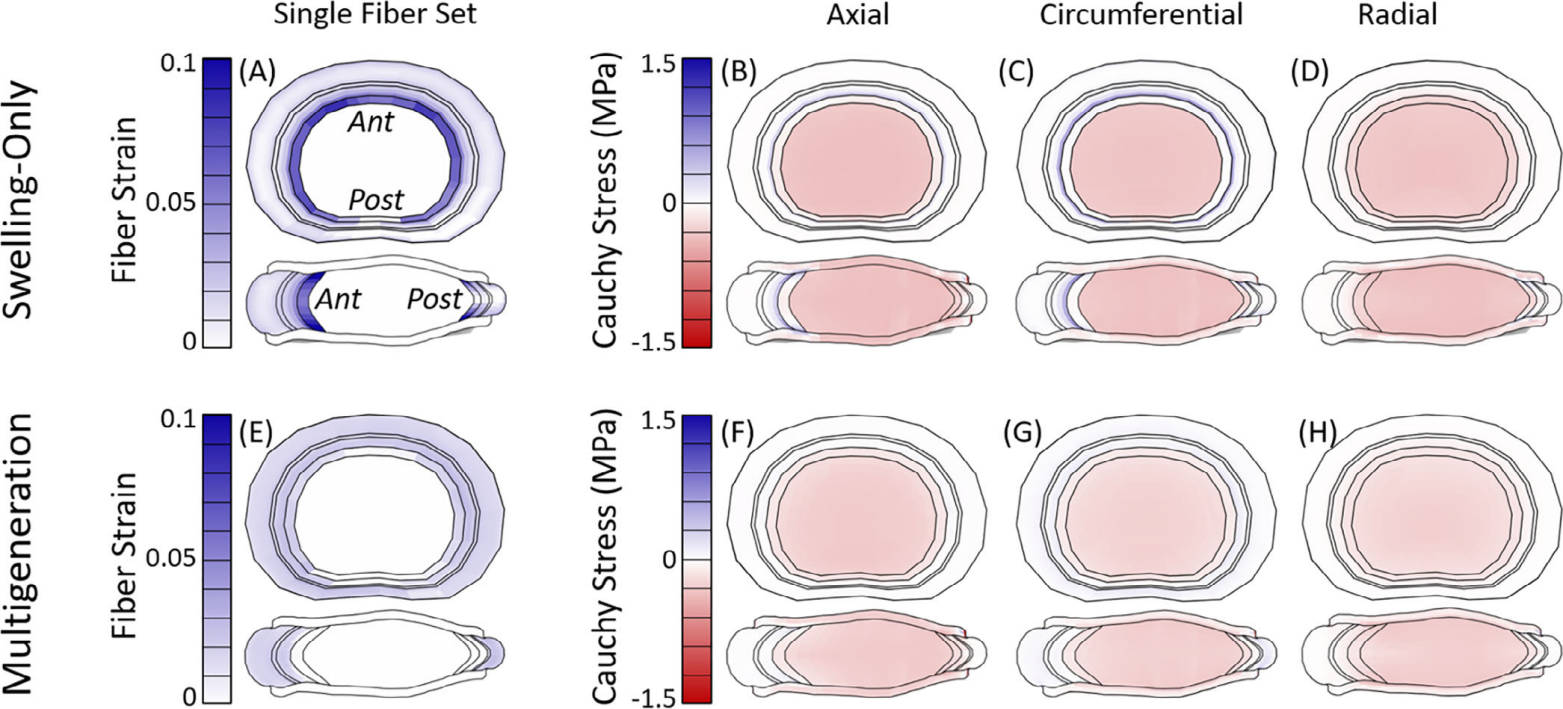
Models after equilibration to 270 N (the preload for all dynamic multiaxial tests). The fiber strain profiles (A, E) were similar for both fiber sets, only one set shown. For the swelling‐only model there was a concentration of fiber strain (A) in the inner AF which caused axial (B) and circumferential (C) Cauchy stress in the same area. For the multigeneration model (Ω = 3°) there was uniform fiber strain (A) throughout the AF and a slight concentration of circumferential stress (G) in the disc posterior

The fiber and disc stress and strain state was evaluated after equilibration to a 270 N load, representing the preload in the muliaxial dynamic tests, for the swelling‐only model (Figure 7A‐D) and multigeneration model with Ω = 3° (Figure 7E‐H). For both models, the fiber strain profiles (Figure 7A,E) were similar between both fiber sets, therefore only one set is shown. The swelling‐only model had a concentration of fiber strain (Figure 7A) in the NP/AF transition and innermost AF which caused high circumferential Cauchy stress in the innermost AF layer (Figure 7C). Similarly, there was high fiber stress in the innermost AF (Figure S4E). The multigeneration model did not have fiber strain concentrations, instead there was uniform fiber strain (Figure 7E) throughout the AF and a uniform circumferential Cauchy stress gradient (Figure 7G). The model also had a uniform fiber stress of ~0.1 MPa (Figure S5G).

### 3.4 Model state: Maximum axial compression

**FIGURE 8:**
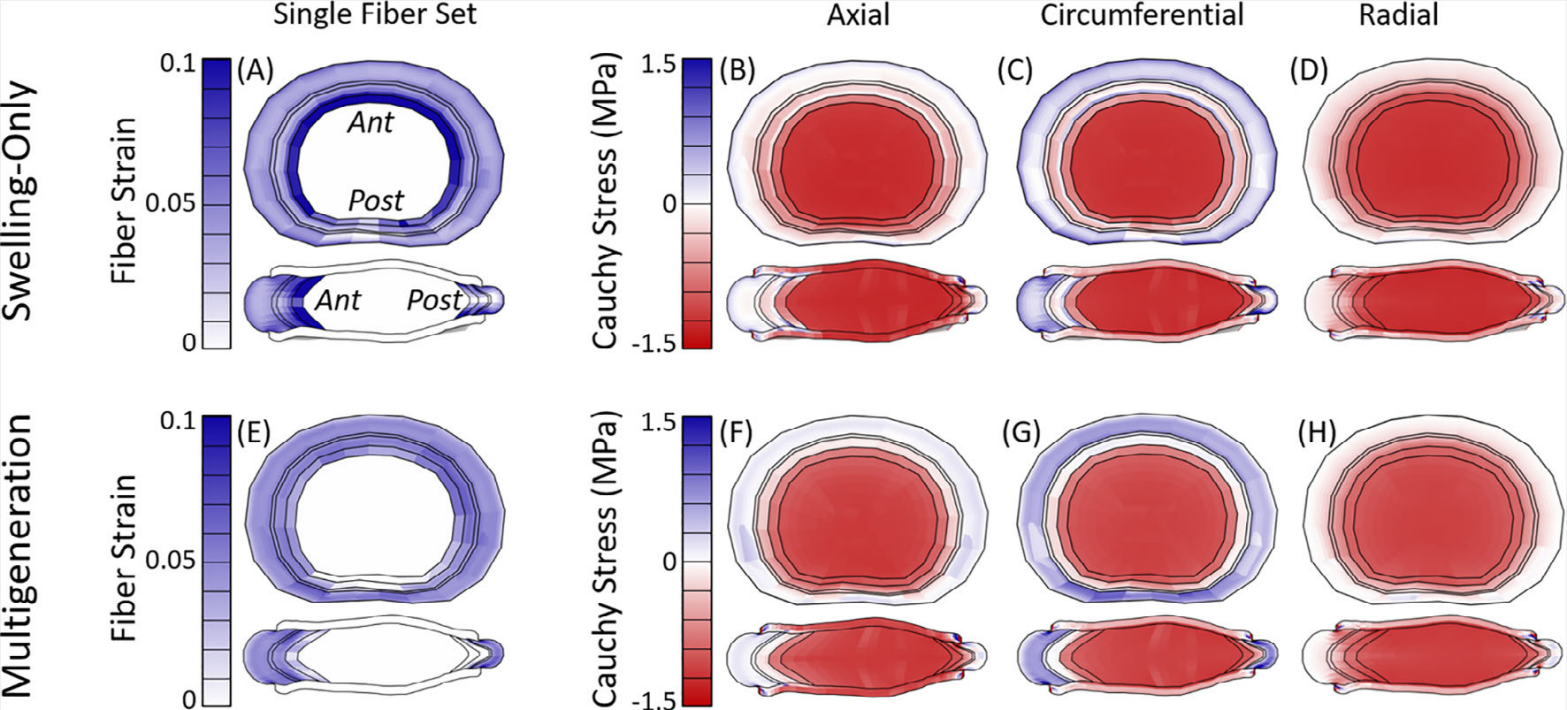
Models at maximum axial compression (from multiaxial dynamic axial compression test case); the fiber strain profiles (A, E) were similar for both fiber sets, only one set shown. For the swelling‐only model, there was a high concentration of fiber strain (A) in the inner most AF and moderate fiber strain through the middle and outer AF. The inner AF was mildly compressed in all directions (B‐D) and the fiber contribution is evident in the circumferential Cauchy stress (C) in the outer AF. The multigeneration model (Ω = 3°) had moderate fiber strain (E) throughout the AF except in the inner, posterior region. There was a moderate, uniform circumferential Cauchy stress (G) in the outer AF

The fiber and disc stress and strain state was evaluated at maximum axial compression from the multiaxial dynamic compression test, for the swelling‐only model (Figure 8A‐D) and multigeneration model with Ω = 3° (Figure 8E‐H). The swelling‐only and multigeneration model profiles are similar to those seen at the end of the 270 N preload, but with greater magnitude as expected due to the increased axial load. The swelling‐only model had a high concentration of fiber strain (Figure 8A) in the inner most AF and moderate fiber strain through the remaining AF. The fiber stress (Figure S6E) followed a similar concentration pattern, with additional concentrations in the middle AF region, particularly at the superior and inferior surfaces. The multigeneration model featured more uniform fiber strain (Figure 8E) and fiber stress (Figure S7E) profiles throughout the AF and the circumferential Cauchy stress (Figure 8C) was highest in the OAF.

### 3.5 Model state: Maximum torsion

**FIGURE 9:**
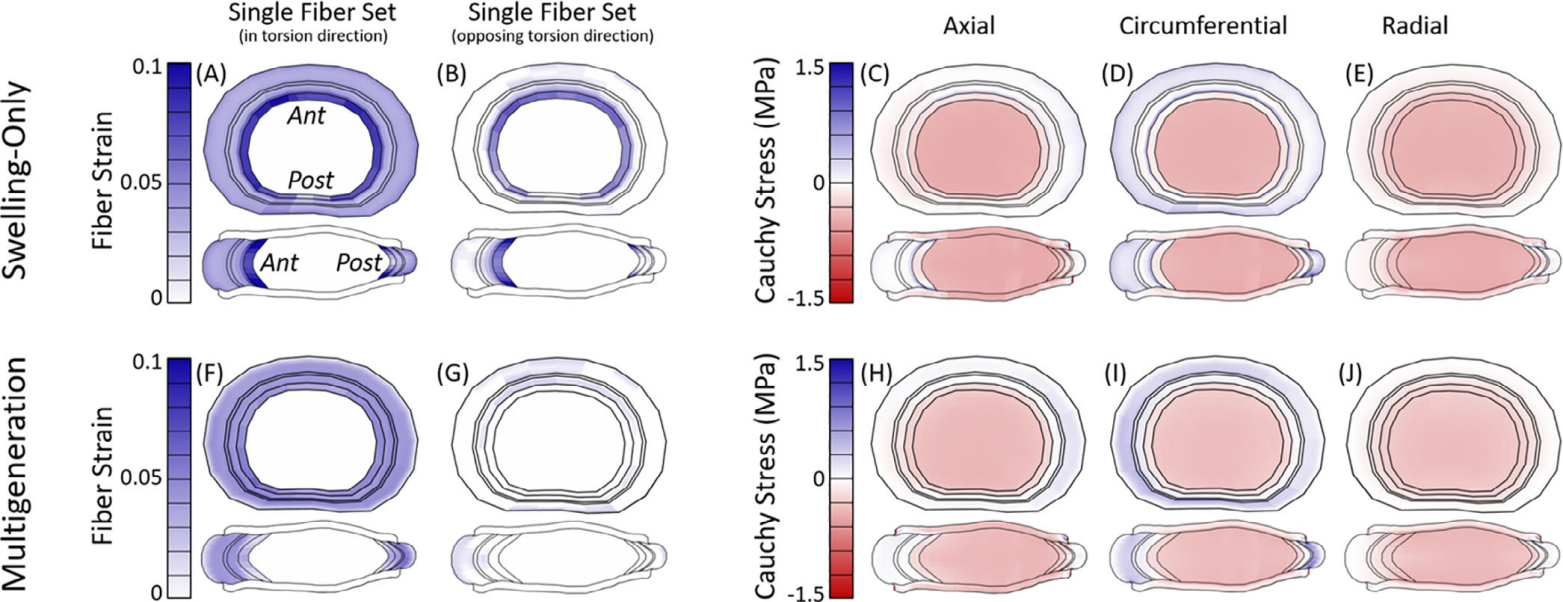
Models at maximum torsional rotation (from multiaxial dynamic torsion test case). For the swelling‐only model, the fibers aligned in the torsion direction (A) experienced moderate fiber strain throughout the AF, and both fiber sets had a high concentration of strain at the NP/AF transition (A, B). There was slight circumferential Cauchy stress (D) in the outer AF. The multigeneration model (Ω = 3°) had moderate fiber strain throughout the AF for fibers aligned in the torsion direction (F) and minimal strain in fibers aligned in the opposing direction (G). The circumferential Cauchy stress (I) was mild in the outer AF and reduced outward

The fiber and disc stress and strain state was evaluated at maximum torsion from the multiaxial dynamic torsion test, for the swelling‐only model (Figure 9A‐E) and multigeneration model with Ω = 3° (Figure 9F‐J). The swelling‐only model at maximum axial torsion experienced highest fiber strain at the NP/AF transition and uniformly moderate fiber strain throughout the remaining AF for the fibers aligned in the direction of torsional rotation (Figure 9A). The fiber set aligned in the opposing direction (Figure 9B) experienced moderate fiber strain at the NP/AF transition; however, fibers in the remaining AF buckled under the torsional rotation and subsequently did not contribute fiber stress. Collectively, the total fiber stress (Figure S9F) was highest in the IAF, and there was a low circumferential Cauchy stress in IAF (Figure 9D). The multigeneration model (Figure 9F,G) once again exhibited uniform fiber contributions throughout the AF, compared to the swelling‐only model (Figure 9A,B). The fiber set aligned in the torsional direction (Figure 9F) experienced moderate fiber strain throughout the AF whereas the fiber set aligned in the opposing direction (Figure 9G) experienced minimal fiber strain in the anterior and posterior regions and the remaining AF fibers buckled. The total fiber stress (Figure S9F) was relatively uniform throughout the AF, with a slight concentration in the middle‐AF posterior region and a similar distribution was also seen in the circumferential Cauchy stress (Figure 9I).

### 3.6 AF fiber contributions across loading scenarios in human disc model

**FIGURE 10:**
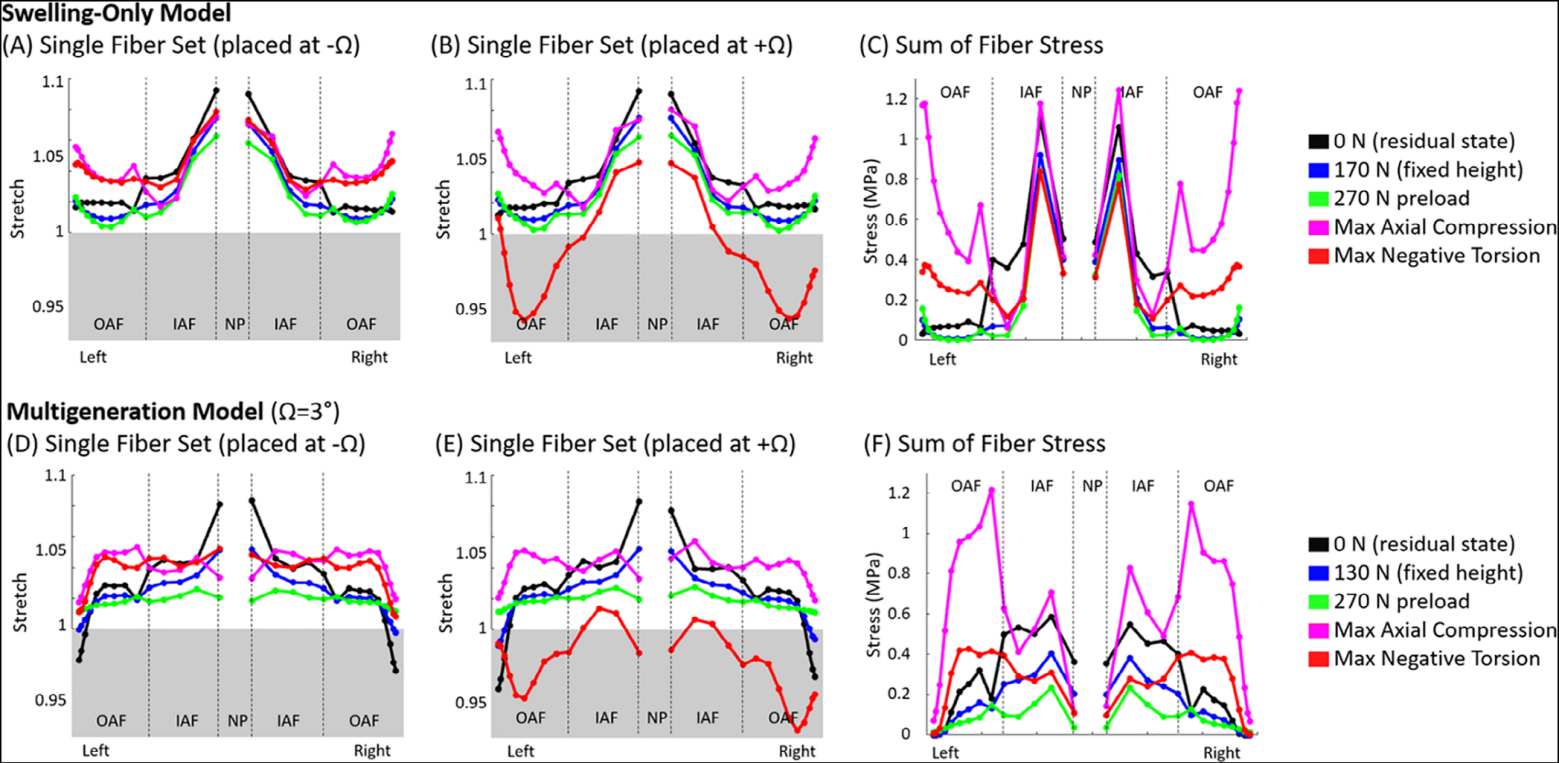
The contribution of AF fibers in the swelling only model (A‐C) and multigeneration model (Ω = 3°) (D‐E) were evaluated across several loading scenarios. A single line of elements at the intersection of the mid‐coronal and mid‐transverse planes were chosen as a representative set. Fiber stretch less than 1 (gray area) indicates the fibers buckled and did not contribute any fiber stress

In order to compare fiber contribution across the disc, the fiber strain and stress in each element from the disc's coronal, mid‐height was plotted across the disc for several loading conditions. Fiber stretch less than one indicates the fibers buckled and, therefore, do not contribute any fiber stress. In the swelling‐only model fiber stretch generally decreases from the outermost OAF toward inner OAF (Figure 10A) whereas the multigen model fiber stretch increases from outermost OAF toward the inner OAF (Figure 10D). Both models tended to have peak fiber stretch at the NP transitional layer, with some cases possessing a secondary peak at the AF transition (Figure 10A,D). The multigeneration model tended to have relatively uniform stretch in the OAF (Figure 10D). For the torsion test case, the models were rotated in the negative direction which results in both models exhibiting significant fiber stretch in the fiber set placed at −Ω (Figure 10A,D) but minimal stretch and much buckling occur in the fiber set placed at +Ω (Figure 10B,E). In the swelling‐only model, fiber stress was highest in the innermost IAF layer and fiber stress generally decreased from the outermost OAF inward (Figure 10C). The multigeneration model also tended to have highest fiber stress in the innermost IAF layer, but fiber stress generally increased from the outermost OAF inward (Figure 10F), unlike the swelling‐only model (Figure 10C). Collectively, the fiber stretch and fiber stress were found to exhibit substantially different profiles between the swelling‐only and multigeneration models.

### 3.7 Bovine disc opening angle

**FIGURE 11:**
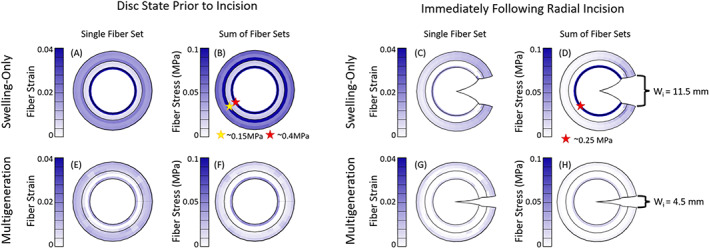
Bovine disc models before and after radial incision. Prior to incision, the swelling‐only model had highly concentrated fiber strain at the AF/NP transition (A) and significant fiber stress at both the inner/outer AF and AF/NP boundaries (B). Following incision, the fiber strain reduced substantially, though a concentration at the AF/NP transition remained (C) and similarly, the fiber stress was still highly concentrated at the AF/NP boundary (D). The multigeneration model prior to incision exhibited a uniform, moderate fiber strain in the OAF and a minimal fiber strain in the IAF (E), there was a slight concentration of fiber stress at the AF/NP transition (F). After incision, the fiber strain (G) and stress (H) reduced with only minimal stress present at the AF/NP transition

The initial opening of the multigeneration model (4.5 mm, Figure 11H) was within the experimental range (4.3 ± 1.8 mm)^18^; the initial opening of the swelling‐only model was greater than expected (11.5 mm, Figure 11D). As was seen in the human disc model, there is a high concentration of both fiber strain (Figure 11A) and fiber stress (Figure 11B) at the AF/NP transition and an additional fiber stress concentration at the inner/outer AF transition. Following radial incision, the fiber strain (Figure 11C) and stress (Figure 11D) in the outer AF reduce dramatically; however the innermost AF layer maintains a high fiber stress concentration. The multigeneration model exhibited relatively uniform fiber strain (Figure 11E) throughout the OAF, with a slight concentration in the innermost AF layer. Similarly, there is a low, uniform fiber stress throughout the OAF with a slight concentration in the innermost AF layer (Figure 11F). The multigeneration model after radial incision exhibits minimal fiber strain, predominantly at the cut plane (Figure 11G) and fiber stress only at the AF/NP transition, but at a substantially reduced magnitude compared to the swelling‐only model (Figure 11D).

## 4 DISCUSSION

This study successfully incorporated residual strain due to swelling and multigeneration fibers in a finite element model and validated the model against uniaxial quasi‐static and multiaxial dynamic tests. We compared a swelling‐only model, with AF residual strain due to swelling, and a multigeneration model, with AF residual strain due to both swelling and inherent AF fiber pre‐strain. We conducted a parametric analysis for the multigeneration model with several parameter combinations to determine the optimal axial displacement (Δ*H* = 0.5 mm) and twist angle (Ω = 3°) for simulating experimental outcomes and by extension, the physiological disc. The models possessed material properties defined by tissue constituent tests and experimental work such that they were not tuned to fit any organ‐scale outcomes investigated. The swelling‐only model lacked the ability to tune fiber residual contributions. The multigeneration model provided a better overall response compared to experiments and had fiber residual strain contributions that could be adjusted independently of the fiber material properties. We were also able to match the residual strain opening gap from bovine experiments with the multigeneration model.^18^

### 4.1 Residual state

The residual state has been investigated in other fibrous soft tissues and multiple underlying mechanisms have been suggested. Fibrous soft tissue residual stress was initially investigated in artery, where it became evident that the unloaded state and stress‐free state of a tissue were not the same.^54^ Later work in disc showed similar outcomes, where a disc cut radially opened as initial residual strains were released.^18^ The sources of residual stress exist in a hierarchy of micro‐, tissue‐, and organ‐scale contributions.^55^ The disc opening is driven by relaxation of tissue‐level residual stress which is initiated during early developmental stages.^18^, ^21^ In the early development of the AF there are angled actin stress fibers present whose initial orientation has a significant impact on the subsequent alignment of the AF fibers.^19^ As the disc grows, the highly organized and aligned fibers are stretched. The AF possesses radial gradients of fiber collagen content, fiber alignment, proteoglycans and water that ultimately result in fully developed discs possessing both inherent fiber pre‐strain as well as swelling‐induced residual strain.^5^, ^8^, ^18^, ^19^, ^21^ In addition to growth, residual contributions in the disc are influenced by the loading state of the tissue, where less axial compression results in less fiber engagement (Figure 7A,E) and greater axial compression results in more fiber engagement (Figure 8A,E). Although the mechanisms may be similar, the use of multigeneration is intended to bring the fibers to a residual strain state similar to that observed in adult discs; our use of multigeneration was not intended to simulate the growth and development of fibers in the disc.

We found modeling the bovine disc incision including residual strain due to swelling alone caused the simulation to overestimate the disc‐opening gap. The multigeneration model, with the inclusion of both swelling and AF fiber residual strain contributions, resulted in an opening gap that matched the experiments.^18^ These outcomes support the necessity of residual strain considerations in disc finite element models and further support that two primary mechanisms of tissue‐level residual strain in the disc are osmotic swelling and pre‐strained AF fibers.

### 4.2 Stress and strain state

We found that the NP was uniformly pressurized in all test cases and consistently exhibited greater Cauchy stress in the swelling‐only model compared to the multigeneration model (Figures 7, 8, 9). When subjected to the 270 N preload, the swelling‐only model exhibited a high circumferential stress in the inner AF, this hoop stress served to contain the NP pressurization (Figure 7A,C). Under the same conditions, the multigeneration model exhibited a uniform fiber contribution throughout the AF, the lack of inner AF concentration was because the fibers are deposited after the swelling simulation and their strain was therefore due largely to the multigeneration placement process, and not by the expansion of the NP during swelling (Figure 7E,G). The low magnitude of fiber contribution is expected under the 270 N load as this preload was chosen to establish a physiologically relevant intradiscal pressure, and under regular physiological conditions we do not expect high fiber engagement. The contribution of fibers was dependent on the discs' axial displacement and twist angle parameters, such that altering either would change the preloaded discs' stress and strain state.

The swelling‐only model consistently possessed high fiber contributions at the AF/NP boundary and had mixed positive and negative circumferential Cauchy stress in the remaining IAF. At maximum axial compression the swelling‐only model had concentrated fiber strain at the AF/NP boundary, though the IAF had both compressive and tensile circumferential stresses, while the OAF exhibited the greatest magnitude of tensile circumferential stress (Figure 8A,C). Under maximum torsional rotation the swelling‐only model exhibited moderate circumferential tension at the AF/NP transition and throughout the OAF. Collectively, the swelling‐only model appears to have an artificial concentration of stress and strain at the AF/NP transition which appears to shield the remaining AF from reasonably contributing to the model's overall mechanical response.

In the multigeneration model, the IAF was consistently in compression, while the outer AF was in moderate tension throughout with reducing magnitude toward the disc's outermost layers (Figure 8E,G). At maximum torsional rotation, the multigeneration model exhibited a moderate, uniform tensile stress distributed throughout the AF which reduced toward the outermost layers, as expected (Figure 9F,I). The multigeneration model does not exhibit inner concentrations and instead repeatedly features a smooth distribution of circumferential fiber contributions throughout the loading scenarios investigated. The lack of inner concentrations is consistent the prior work in artery, where inclusion of residual stress significantly reduced inner stress concentrations of the vessel following loading.^54^ The uniform fiber contribution enabled by the multigeneration model with an adequate twist angle (Ω ≥ 3°) seems necessary for recapitulating experimental outcomes in torsion; however, the increase in fiber contribution throughout the AF simultaneously worsens the model's ability to match stress relaxation outcomes.

### 4.3 Parametric analysis of the multigeneration model

We conducted a parametric analysis of the multigeneration model parameters (axial displacement (Δ*H*) and twist angle (Ω)) because the model geometry at the time of fiber placement impacts the fiber pre‐strain imposed and subsequently, the model's performance in test cases. Ultimately, across all seven test cases, the multigeneration model that provided the best overall fit to experimental data was the model with Δ*H* = 0.5 mm and Ω = 3°. When a larger twist angle (Ω = 4°) was used, the stress relaxation response was poor, due to the large initial load that propagated throughout the subsequent relaxation (Figure 5C). When a smaller twist angle (Ω = 2°) was imposed, the fit to torsion was poor because the model did not possess sufficient fiber contribution to support the disc in torsional rotation (Figure 5E). Therefore, we selected a twist angle of 3° as a compromise between these effects. In addition, through parametric analysis we found that an axial displacement of 0.5 mm was necessary to recapitulate experimental outcomes, particularly for quasi‐static uniaxial slow ramp and creep tests (Figures S2 and S3).

### 4.4 Material property selection

An advantage of our disc model is that the material properties are defined based on tissue testing and were not tuned to fit the organ‐scale outcomes. Moreover, we included radial variation between the inner and outer AF and included transitional regions between the AF sections and the NP. The matrix properties and tissue permeability were determined from confined compression tissue testing (Table 1).^6^, ^13^ The fixed charge density of all tissues, essential for simulating appropriate swelling, was determined from proteoglycan assessment in each tissue (Table 1).^13^, ^21^ The fiber properties were based on tensile tissue testing^31^, ^32^, ^34^ and fiber angles were from optical microscope assessment (Table 1).^5^ Our material property definitions are rigorously rooted in experimental tissue tests, such that each tissue represented in our model accurately mimics the mechanical contribution of the physiological tissues and collectively enables our organ‐scale model to optimally represent the whole, physiological disc.

We included radial variation in the material properties; however, we do not include regional circumferential differences between the anterior, posterior, and lateral regions, such as higher proteoglycan content in the posterior region.^8^ There is also contradictory information in the literature as to whether there are regional differences in fiber angle.^5^, ^33^ Optical microscope analysis of AF tissue found no circumferential differences, only substantial radial variation in fiber angle^5^; however, later experimental work utilized surface marking and digital imaging which revealed regional variation in fiber angle.^56^ In addition, mathematical modeling suggested that the inner posterior region of the disc has substantially higher fiber angle than through the rest of the AF.^33^ While our model does not possess regional variation, we account for radial differences in material properties which are greater in magnitude.

### 4.5 Limitations

The organ‐scale size and shape of the disc under load was generally reasonable with the exception of excess posterior protrusion (see Figure 8). A similar model previously used by our lab found that the model showed the greatest difference from the MRI measurements in the posterior region where the model had excess radial strain compared to the MRI‐based strain analysis.^48^ The underlying cause was suggested to be elongated rectangular elements in posterior unintentionally causing small discontinuities in the fiber distribution.^48^ The posterior protrusion might be corrected in future work by refining the mesh in that region, though in the present cases it does not impose unreasonable stress or strain concentrations nor does it impair the model's gross response.

We were unable to explicitly define fiber pre‐strain in our biphasic model in FEBio, which limits the ability to control the pre‐strain imposed on the AF fibers. We considered multiple placement methods to generate fiber pre‐strain and ultimately found that the multigeneration feature with organ‐scale axial displacement and rotation created an adequate deformation state for fiber placement to produce pre‐strain. This technique to impose fiber pre‐strain was not intended to represent physiological loading for either the human or bovine discs, it was purely to establish pre‐strained fibers in the AF. Fiber placement by this method was limited as all fibers were placed with the disc in the same organ‐scale deformation state; however, explicit ability to impose particular pre‐strain would greatly improve the control and precision of fiber pre‐strain. FEBio was previously used to implement explicitly defined fiber pre‐strain in a ligament model,^57^ but we were unable to use their methods here due to the complexity of our biphasic model. Expansion of FEBio's pre‐strain fiber method in future versions would allow for pre‐strained fiber contributions to be more readily utilized in fibrous soft tissue models.

## 5 CONCLUSION/RECOMMENDATION

In this study, we successfully incorporated residual strain due to swelling and multigeneration fibers in a finite element model of intervertebral disc and validated human disc models against uniaxial quasi‐static and multiaxial dynamic tests. The swelling‐only model was within the confidence interval for all human disc outcomes, though it overestimated the opening of the incised bovine model. The multigeneration model provided better human disc model responses, closer to the experimental mean and had a bovine model opening angle within the experimental range. The use of multigeneration allowed for the inclusion of inherently pre‐strained fibers in AF, which produced uniform fiber contribution throughout the AF and parametric analysis found a twist angle of 3° and axial displacement of 0.5 mm were necessary for optimizing the multigeneration model outcomes. The inclusion of swelling and fiber‐induced residual strain in the multigeneration model was necessary for achieving a physiological residual strain state and for replicating disc mechanical behavior across uniaxial quasi‐static and multiaxial dynamic test cases.

## 6 SUPPLEMENTAL

### 6.1 Previous model

Our lab previously developed a disc finite element model, detailed in Reference 4. The model was validated against uniaxial quasi‐static slow ramp, creep, and stress relaxation, but when expanded to multiaxial dynamic tests it was unable to recapitulate experimental outcomes (Figure S1).

### 6.2 Parametric analysis of multigeneration model: Axial displacement

The normalized mean square error (NMSE) was calculated as explained in the main manuscript Methods 2.4.1) Nonlinear Disc Response. Variations in axial displacement majorly impacted the uniaxial, quasi‐static outcomes in creep and stress relaxation (ΔNMSE > 0.35) and mildly impacted slow ramp and dynamic torsion (0.25 < ΔNMES < 0.15) (Figure S3). Dynamic multiaxial tests axial compression, bending, and flexion were minimally impacted by the axial displacement parameter (ΔNMSE < 0.1) (Figure S3). An axial displacement of 0.5 mm to achieve a fiber placement height of 11.5 mm was deemed necessary based largely on the slow ramp and creep outcomes. A fiber placement height of 11 mm over‐estimated compressive displacement and a fiber placement height of 12 mm underestimated compressive displacement in both tests (Figures S2 and S3). Furthermore, fiber placement height of 11.5 mm was necessary to compromise between the stress relaxation and torsion outcomes, where stress relaxation response improved with increasing fiber placement height but the torsion response improved with decreasing fiber placement height (Figures S2 and S3).

### 6.3 Human disc fiber and model stress and strain states

In addition to the fiber strain and Cauchy stresses in the main paper figures, the fiber stress as well as axial, circumferential and radial Lagrange strains were also evaluated for the swelling‐only model at the end of preload (Figure S4), maximum axial compression (Figure S6), and maximum torsion (Figure S8) and for the multigeneration model at the end of preload (Figure S5), maximum axial compression (Figure S7), and maximum torsion (Figure S9).

### 6.4 Bovine disc model stress and strain states

The axial, circumferential and radial stress and strain were quantified for the bovine disc models preceding radial incision and immediately following the incision (Figure S10).

### 6.5 Calculation of the fiber strain and stress

AF fiber strain and stress were quantified to evaluate their contribution to the gross mechanical behavior of the disc. This was accomplished on an element‐by‐element basis within the layers which had residual stress from AF fibers. Fibers were aligned by specification of local [1, 2, 5] axis (Figure S11, see FEBio manual for further details), each element had node positions (x, y, z) which were used to determine the $\vec{a}$ and $\vec{d}$ directions at the start of the simulation (Figure S11):

$$\vec{a}=n2\left(x,y,z\right)-n1\left(x,y,z\right)\;\text{and}\;\vec{d}=n5\left(x,y,z\right)-n1\left(x,y,z\right)$$

These initial directions were used to calculate a set of orthonormal fiber vectors:

$$e_{1}=\frac{a}{\left\|a\right\|},e_{2}=e_{3}\;x\;e_{1},e_{3}=\frac{a\;x\;d}{\left\|a\;x\;d\right\|}$$

Which were then compiled into a tensor, one for each element:

$$T_{x,B}=\left[\begin{array}{c} e1_{x}\;\\ e1_{y}\\ e1_{z} \end{array}\begin{array}{c} e2_{x}\\ \;e2_{y}\\ \;e2_{z} \end{array}\begin{array}{c} \;e3_{x}\\ \;e3_{y}\\ \;e3_{z} \end{array}\right]$$

The directional vector for the fibers in terms of global coordinates was then determined for time zero:

$$v_{f\;\text{global}}=T_{x,B}∙v_{f\;\text{local}}\;\text{where}\;v_{f\;\text{local}}=\left[\cos\left(\theta \right)\;\sin\left(\theta \right)\;0\right]$$

In order to calculate fiber stretch, it was necessary that the reference time be the multigeneration time, not time zero. To get the global position vector at the fiber deposition time:

$$v_{f\;\text{global},\text{multigen}}=F\left(x,u\right)∙v_{f\;\text{global}}$$

where *F*(*x*, *u*) was the deformation gradient at the time of deposition (*u*) with respect to time zero. With the global directional vector at deposition time known, it was necessary to determine the deformation gradient at the time of interest (*t*) with respect to the deposition time:

$$F\left(x,t\right)F{\left(x,u\right)}^{-1}=F^{u}\left(x,t\right)$$

Now with the appropriate deformation gradient, the Right Cauchy‐Green Strain was calculated:

$$C_{\text{multigen}}={\left(F^{u}\left(x,t\right)\right)}^{T}F^{u}\left(x,t\right)$$

and the fiber stretch was determined:

$$\lambda_{n}=\sqrt{v_{f\;\text{global},\text{multigen}}∙C_{\text{multigen}}∙v_{f\;\text{global},\text{multigen}}}$$

The fiber strain was defined as fiber stretch minus one such that:

$$\varepsilon_{n}=\lambda_{n}-1$$

In addition to fiber stretch, the fiber stress was also quantified:

$$\sigma \left(\lambda_{n}\right)=\frac{d\Psi_{n}\left(\lambda_{n}\right)}{d\lambda_{n}}=\left\{\begin{array}{c} 0\;\lambda_{n}^{2}<1\;\\ \frac{E_{f}}{2\left(\beta_{f}-1\right)}\lambda_{n}^{2}{\left(\lambda_{n}^{2}-1\right)}^{\beta_{f}-1}\;1\leq \lambda_{n}^{2}\leq \lambda_{0}^{2}\;\\ \frac{\lambda_{n}^{2}}{\lambda_{0}}\frac{E_{f}}{2\left(\beta_{f}-1\right)}\left(1-\frac{1}{\lambda_{0}^{2}}\right)+E_{f}\lambda_{n}\left(\frac{\lambda_{n}}{\lambda_{0}}-1\right)\;\lambda_{0}^{2}<\lambda_{n}^{2}\; \end{array}\right\}$$

where *λ*_*n*_ was the fiber stretch previously calculated, and all other properties including the fiber modulus (*E*_*f*_), toe‐region power law exponential (*β*_*f*_), and transition stretch (*λ*_*o*_) were specified for each constituent (Table 1).

## CONFLICT OF INTEREST

The authors have no conflicts of interest to report.

## AUTHOR CONTRIBUTIONS

All contributed to project concept. HRN, JFD, JMP performed modeling work. HRN, JMP, DME prepared manuscript. All contributed to manuscript revisions.

## Supporting information


**Figure S1** The Jacobs+2014 model was not able to recapitulate experimental outcomes in dynamic axial compression (A) and torsion (B). C, NMSE values closer to zero indicate a model response closer to the experimental mean and NMSE value greater than 1 indicate the model response was outside the experimental 95% confidence interval. D, The model equilibrated to a 50 N load had reasonable height compared to MRI data and had mild excess bulge (E).

**Figure S2** Experimental data ±95% confidence interval is shown with model outcomes for the multigeneration models with Ω = 3° and variable axial displacement (Δ*H*). The model with Δ*H* = 0.5 mm was optimal for uniaxial quasi‐static slow ramp (A) and creep (B) outcomes. For uniaxial quasi‐static stress relaxation (C), the model with Δ*H* = 1 mm was best. There was minimal variation between the multigeneration model outcomes in multiaxial dynamic axial compression (D), bending (F), and flexion (G). The model with Δ*H* = 0 mm offered the best torsion response (E).

**Figure S3** A, Normalized mean square error (NMSE) values are shown for the multigeneration model with varying axial displacement (Δ*H*) with twist angle Ω = 3°. NMSE values closer to zero indicate a model response closer to the experimental mean. Axial displacement Δ*H* = 0.5 mm was necessary to optimize slow ramp and creep outcomes. Stress relaxation was best at Δ*H* = 1.0 mm while torsion was best at Δ*H* = 0.0 mm. All model responses were reasonable in axial compression, bending, and torsion tests. B and C, Disc height and bulge from the models equilibrated to a 50 N load compared to MRI data of discs under a 50 N load. B, All models were within expected height limits. C, All models are in the upper range of disc bulge.

**Figure S4** The stress and strain state of the swelling‐only model following equilibration to a 270 N preload, a relevant pre‐test state for the multiaxial dynamic tests. The fiber strain profile was similar for both fiber sets, only one set shown. Fiber strain and Cauchy stresses (A, F‐H) are identical to those shown in Figure 7A‐D, respectively.

**Figure S5** The stress and strain state of the multigeneration model (Ω = 3°) following equilibration to a 270 N preload, a relevant pre‐test state for the multiaxial dynamic tests. The fiber strain profile was similar for both fiber sets, only one set shown. Fiber strain and Cauchy stresses (A, F‐H) are identical to those shown in Figure 7E‐H, respectively.

**Figure S6** The stress and strain state of the swelling‐only model at maximum compression from the multiaxial dynamic axial compression test. The fiber strain profile was similar for both fiber sets, only one set shown. Fiber strain and Cauchy stresses (A, F‐H) are identical to those shown in Figure 8A‐D, respectively.

**Figure S7** The stress and strain state of the multigeneration model (Ω = 3°) at maximum compression from the multiaxial dynamic axial compression test. The fiber strain profile was similar for both fiber sets, only one set shown. Fiber strain and Cauchy stresses (A, F‐H) are identical to those shown in Figure 8E‐H, respectively.

**Figure S8** The stress and strain state of the swelling‐only model at maximum torsion from the multiaxial dynamic torsion test. Fiber strains and Cauchy stresses (A‐B, F‐H) are identical to those shown in Figure 9A‐E, respectively.

**Figure S9** The stress and strain state of the multigeneration model (Ω = 3°) at maximum torsion from the multiaxial dynamic torsion test. Fiber strains and Cauchy stresses (A‐B, F‐H) are identical to those shown in Figure 9F‐J, respectively.

**Figure S10** The bovine disc model immediately before (A‐C, G‐I) and following radial incision (D‐F, J‐L) for the swelling‐only model (A‐F) and multigeneration model (G‐L).

**Figure S11** Disc model with anterior (A), posterior (P), superior (S), left (L) and right (R) directions labeled. A schematic of a single element with local node numbers and directional vectors 'a' and 'd' shown for a local material axis definition of [1, 2, 5] for more details see FEBio user manual.

## References

[1] Ehlers W , Karajan N , Markert B . An extended biphasic model for charged hydrated tissues with application to the intervertebral disc. Biomech Model Mechanobiol. 2009;8(3):233‐251. 10.1007/s10237-008-0129-y. 18661285

[2] Frijns A , Huyghe J , Janssen J . A validation of the Quadriphasic mixture theory for intervertebral disc tissue. Int J Eng Sci. 1997;35(15):1419‐1429.

[3] Iatridis JC , Setton LA , Foster RJ , Rawlins BA , Weidenbaum M , Mow VC . Degeneration affects the anisotropic and nonlinear behaviors of human anulus fibrosus in compression. J Biomech. 1998;31(6):535‐544. 10.1016/S0021-9290(98)00046-3. 9755038

[4] Jacobs NT , Cortes DH , Peloquin JM , Vresilovic EJ , Elliott DM . Validation and application of an intervertebral disc finite element model utilizing independently constructed tissue‐level constitutive formulations that are nonlinear, anisotropic, and time‐dependent. J Biomech. 2014;47(11):2540‐2546. 10.1016/j.jbiomech.2014.06.008. 24998992 PMC4366133

[5] Cassidy J , Hiltner A , Baer E . Hierarchical structure of the intervertebral disc. Connect Tissue Res. 1989;23(1):75‐88. 2632144 10.3109/03008208909103905

[6] Cortes DH , Jacobs NT , DeLucca JF , Elliott DM . Elastic, permeability and swelling properties of human intervertebral disc tissues: a benchmark for tissue engineering. J Biomech. 2014;47(9):2088‐2094. 10.1016/j.jbiomech.2013.12.021. 24438768 PMC4047194

[7] Dreischarf M , Shirazi‐Adl A , Arjmand N , Rohlmann A , Schmidt H . Estimation of loads on human lumbar spine: a review of in vivo and computational model studies. J Biomech. 2016;49(6):833‐845. 10.1016/j.jbiomech.2015.12.038. 26873281

[8] Iatridis JC , MacLean JJ , O'Brien M , Stokes IAF . Measurements of proteoglycan and water content distribution in human lumbar intervertebral discs. Spine. 2007;32(14):1493‐1497. 10.1097/BRS.0b013e318067dd3f. 17572617 PMC3466481

[9] Yang B , O'Connell GD . Effect of collagen fibre orientation on intervertebral disc torsion mechanics. Biomech Model Mechanobiol. 2017;16(6):2005‐2015. 10.1007/s10237-017-0934-2. 28733922

[10] Johannessen W , Elliott DM . Effects of degeneration on the biphasic material properties of human nucleus pulposus in confined compression. Spine. 2005;30(24):724‐729. 10.1097/01.brs.0000192236.92867.15. 16371889

[11] Tavakoli J , Elliott DM , Costi JJ . Structure and mechanical function of the inter‐lamellar matrix of the annulus fibrosus in the disc. J Orthop Res. 2016;34(8):1307‐1315. 10.1002/jor.23306. 27208689

[12] Wu HC , Yao RF . Mechanical behavior of the human annulus fibrosus. J Biomech. 1976;9(1):1‐7. 10.1016/0021-9290(76)90132-9. 1249075

[13] DeLucca JF , Cortes DH , Jacobs NT , Vresilovic EJ , Duncan RL , Elliott DM . Human cartilage endplate permeability varies with degeneration and intervertebral disc site. J Biomech. 2016;49(4):550‐557. 10.1016/J.JBIOMECH.2016.01.007. 26874969 PMC4779374

[14] MacLean JJ , Owen JP , Iatridis JC . Role of endplates in contributing to compression behaviors of motion segments and intervertebral discs. J Biomech. 2007;40(1):55‐63. 10.1016/j.jbiomech.2005.11.013. 16427060 PMC2757141

[15] Malandrino A , Lacroix D , Hellmich C , Ito K , Ferguson SJ , Noailly J . The role of endplate poromechanical properties on the nutrient availability in the intervertebral disc. Osteoarthr Cartil. 2014;22:1053‐1060. 10.1016/j.joca.2014.05.005. 24857972

[16] Sampson SL , Sylvia M , Fields AJ . Effects of dynamic loading on solute transport through the human cartilage endplate. J Biomech. 2019;83:273‐279. 10.1016/j.jbiomech.2018.12.004. 30554819 PMC6326858

[17] Duclos SE , Michalek AJ . Residual strains in the intervertebral disc annulus fibrosus suggest complex tissue remodeling in response to in‐vivo loading. J Mech Behav Biomed Mater. 2017;68(February):232‐238. 10.1016/j.jmbbm.2017.02.010. 28232297

[18] Michalek A , Gardner‐Morse M , Iatridis J . Large residual strains are present in the intervertebral disc annulus Fibrosus in the unloaded state. J Biomech. 2012;45(7):1‐12. 22342138 10.1016/j.jbiomech.2012.01.042 PMC3327823

[19] Hayes AJ , Benjamin M , Ralphs JR . Role of Actin stress fibres in the development of the intervertebral disc: cytoskeletal control of extracellular matrix assembly. Dev Dyn. 1999;215(3):179‐189. 10.1002/(SICI)1097-0177(199907)215:3<179::AID-AJA1>3.0.CO;2-Q. 10398529

[20] Sharabi M , Wade K , Haj‐Ali R . Mechanical Role of Collagen Fibers in the Intervertebral Disc; 2019.

[21] Cortes DH , Elliott DM . Extra‐fibrillar matrix mechanics of annulus fibrosus in tension and compression. Biomech Model Mechanobiol. 2012;11(6):781‐790. 10.1007/s10237-011-0351-x. 21964839 PMC3500513

[22] Galbusera F , Schmidt H , Noailly J , et al. Comparison of four methods to simulate swelling in poroelastic finite element models of intervertebral discs. J Mech Behav Biomed Mater. 2011;4(7):1234‐1241. 10.1016/j.jmbbm.2011.04.008. 21783132

[23] Gu WY , Lai WM , Mow VC . A mixture theory for charged‐hydrated soft tissues containing ivjulti‐electrolytes: passive transport and swelling behaviors. J Biomech Eng. 1998;120(2):169‐180. 10.1115/1.2798299. 10412377

[24] Iatridis JC , Laible JP , Krag MH . Influence of fixed charge density magnitude and distribution on the intervertebral disc: applications of a poroelastic and chemical electric (PEACE) model. J Biomech Eng. 2003;125(February):12‐24. 10.1115/1.1533804. 12661193

[25] Lai WM , Hou JS , Mow VC . A triphasic theory for the swelling and deformation behaviors of articular cartilage. J Biomech Eng. 1991;113(3):245‐258. http://www.ncbi.nlm.nih.gov/pubmed/1921350. 1921350 10.1115/1.2894880

[26] Schmidt H , Bashkuev M , Galbusera F , Wilke HJ , Shirazi‐Adl A . Finite element study of human lumbar disc nucleus replacements. Comput Methods Biomech Biomed Eng. 2014;17(16):1762‐1776. 10.1080/10255842.2013.766722. 23477684

[27] Schroeder Y , Wilson W , Huyghe JM , Baaijens FPT . Osmoviscoelastic finite element model of the intervertebral disc. Eur Spine J. 2006;15(SUPPL. 3):361‐371. 10.1007/s00586-006-0110-3. PMC2335381 16724211

[28] Wilson W , van Donkelaar C , Huyghe J . A comparison between mechano‐electrochemical and biphasic swelling theories for soft hydrated tissues. J Biomech Eng. 2005;127(1):158‐165. 10.1115/1.1835361. 15868798

[29] Yang B , O'Connell GD . Intervertebral disc swelling maintains strain homeostasis throughout the annulus fibrosus: a finite element analysis of healthy and degenerated discs. Acta Biomater. 2019;100:61‐74. 10.1016/j.actbio.2019.09.035. 31568880

[30] Maas SA , Ellis BJ , Ateshian GA , Weiss JA . FEBio: finite elements for biomechanics. J Biomech Eng. 2012;134(1):011005. 10.1115/1.4005694. 22482660 PMC3705975

[31] Elliott DM , Setton LA . Anisotropic and inhomogeneous tensile behavior of the human Anulus Fibrosus: experimental measurement and material model predictions. J Biomech Eng. 2001;123(3):256‐263. 10.1115/1.1374202. 11476369

[32] Jacobs NT , Cortes DH , Vresilovic EJ , Elliott DM . Biaxial tension of fibrous tissue: using finite element methods to address experimental challenges arising from boundary conditions and anisotropy. J Biomech Eng. 2013;135(2):1‐10. 10.1115/1.4023503. PMC3705970 23445049

[33] Michalek AJ . A growth‐based model for the prediction of fiber angle distribution in the intervertebral disc annulus fibrosus. Biomech Model Mechanobiol. 2019;18(5):1363‐1369. 10.1007/s10237-019-01150-4. 30980210

[34] O'Connell GD , Guerin HL , Elliott DM . Theoretical and uniaxial experimental evaluation of human annulus fibrosus degeneration. J Biomech Eng. 2009;131(11):111007. 10.1115/1.3212104. 20353258 PMC3424515

[35] Beckstein JC , Sen S , Schaer TP , Vresilovic EJ , Elliott DM . Comparison of animal discs used in disc research to human lumbar disc: axial compression mechanics and glycosaminoglycan content. Spine. 2008;33(6):166‐173. 10.1097/BRS.0b013e318166e001. 18344845

[36] Bezci SE , Werbner B , Zhou M , et al. Radial variation in biochemical composition of the bovine caudal intervertebral disc. Jor Spine. 2019;2(3):1‐13. 10.1002/jsp2.1065. PMC6764789 31572982

[37] Demers CN , Antoniou J , Mwale F . Value and limitations of using the bovine tail as a model for the human lumbar spine. Spine. 2004;29(24):2793‐2799. 10.1097/01.brs.0000147744.74215.b0. 15599281

[38] Showalter BL , Beckstein JC , Martin JT , et al. Comparison of animal discs used in disc research to human lumbar disc: torsion mechanics and collagen content. Spine. 2012;37(15):E900‐E907. 10.1097/BRS.0b013e31824d911c. 22333953 PMC3377819

[39] Peloquin JM , Yoder JH , Jacobs NT , et al. Human L3L4 intervertebral disc mean 3D shape, modes of variation, and their relationship to degeneration. J Biomech. 2014;47(10):2452‐2459. 10.1016/j.jbiomech.2014.04.014. 24792581 PMC4115453

[40] Holmes MH , Mow VC . The nonlinear characteristics of soft gels and hydrated connective tissues in ultrafiltration. J Biomech. 1990;23(11):1145‐1156. 10.1016/0021-9290(90)90007-P. 2277049

[41] Finley SM , Brodke DS , Spina NT , DeDen CA , Ellis BJ . FEBio finite element models of the human lumbar spine. Comput Methods Biomech Biomed Eng. 2018;21(6):444‐452. 10.1080/10255842.2018.1478967. 30010415

[42] Ateshian GA , Warden WH , Kim JJ , Grelsamer RP , Mow VC . Finite deformation biphasic material properties of bovine articular cartilage from confined compression experiments. J Biomech. 1997;30(11–12):1157‐1164. 10.1016/S0021-9290(97)85606-0. 9456384

[43] Ateshian GA . Anisotropy of fibrous tissues in relation to the distribution of tensed and buckled fibers. J Biomech Eng. 2007;129(2):240‐249. 10.1115/1.2486179. 17408329 PMC2805028

[44] Hou C , Ateshian GA . Gauss‐Kronrod‐trapezoidal integration scheme for modeling biological tissues with continuous fiber distributions. Comput Methods Biomech Biomed Eng. 2016;19(8):889‐893. 10.1080/10255842.2015.1075518 PMC4807401 26291492

[45] Goel VK , Ramirez SA , Kong W , Gilbertson LG . Cancellous bone young's modulus variation within the vertebral body of a ligamentous lumbar spine—application of bone adaptive remodeling concepts. J Biomech Eng. 1995;117(3):266‐272. 10.1115/1.2794180. 8618378

[46] Mow VC , Kuei SC , Lai WM , Armstrong CG . Biphasic creep and stress relaxation of articular cartilage in compression: theory and experiments. J Biomech Eng. 1980;102(1):73‐84. 10.1115/1.3138202. 7382457

[47] O'Connell GD , Jacobs NT , Sen S , Vresilovic EJ , Elliott DM . Axial creep loading and unloaded recovery of the human intervertebral disc and the effect of degeneration. J Mech Behav Biomed Mater. 2011;4(7):933‐942. 10.1016/j.jmbbm.2011.02.002. 21783103 PMC3143379

[48] Showalter BL , DeLucca JF , Peloquin JM , et al. Novel human intervertebral disc strain template to quantify regional three‐dimensional strains in a population and compare to internal strains predicted by a finite element model. J Orthop Res. 2016;34(7):1264‐1273. 10.1002/jor.23137. 26694516 PMC5244430

[49] DeLucca JF , Amin D , Peloquin JM , Vresilovic EJ , Costi JJ , Elliott DM . Off‐axis response due to mechanical coupling across all six degrees of freedom in the human disc. JOR Spine. 2019;2(1):e1047. 10.1002/jsp2.1047. 31463461 PMC6686826

[50] Amin DB , Sommerfeld D , Lawless IM , Stanley RM , Ding B , Costi JJ . Effect of degeneration on the six degree of freedom mechanical properties of human lumbar spine segments. J Orthop Res. 2016;34(8):1399‐1409. 10.1002/jor.23334. 27291789

[51] Yoder JH , Peloquin JM , Song G , et al. Internal three‐dimensional strains in human intervertebral discs under axial compression quantified noninvasively by magnetic resonance imaging and image registration. J Biomech Eng. 2014;136(11):1‐9. 10.1115/1.4028250. PMC4181341 25109533

[52] O'Connell GD , Vresilovic EJ , Elliott DM . Comparison of animals used in disc research to human lumbar disc geometry. Spine. 2007;32(3):328‐333. 10.1097/01.brs.0000253961.40910.c1. 17268264

[53] Mengoni M , Kayode O , Sikora SNF , Zapata‐Cornelio FY , Gregory DE , Wilcox RK . Annulus fibrosus functional extrafibrillar and fibrous mechanical behaviour: experimental and computational characterisation. R Soc Open Sci. 2017;4(8):105–123. 10.1098/rsos.170807. PMC5579130 28879014

[54] Chuong CJ , Fung YC . On residual stresses in arteries. J Biomech Eng. 1986;108:189‐192. 3079517 10.1115/1.3138600

[55] Lanir Y . Mechanisms of residual stress in soft tissues. J Biomech Eng. 2009;131(4):1‐5. 10.1115/1.3049863. 19275448

[56] Holzapfel GA , Schulze‐Bauer CAJ , Feigl G , Regitnig P . Single lamellar mechanics of the human lumbar anulus fibrosus. Biomech Model Mechanobiol. 2005;3(3):125‐140. 10.1007/s10237-004-0053-8. 15778871

[57] Weiss JA , Gardiner JC , Ellis BJ , Lujan TJ , Phatak NS . Three‐dimensional finite element modeling of ligaments: technical aspects. Med Eng Phys. 2005;27(10):845‐861. 10.1016/j.medengphy.2005.05.006. 16085446

