# Supplementary figures and images for: Multiaxial validation of a finite element model of the intervertebral disc with multigenerational fibers to establish residual strain
Source: JOR Spine. 2021 Mar 21;4(2):e1145. doi: 10.1002/jsp2.1145 (PMC8313175; doi:10.1002/jsp2.1145)

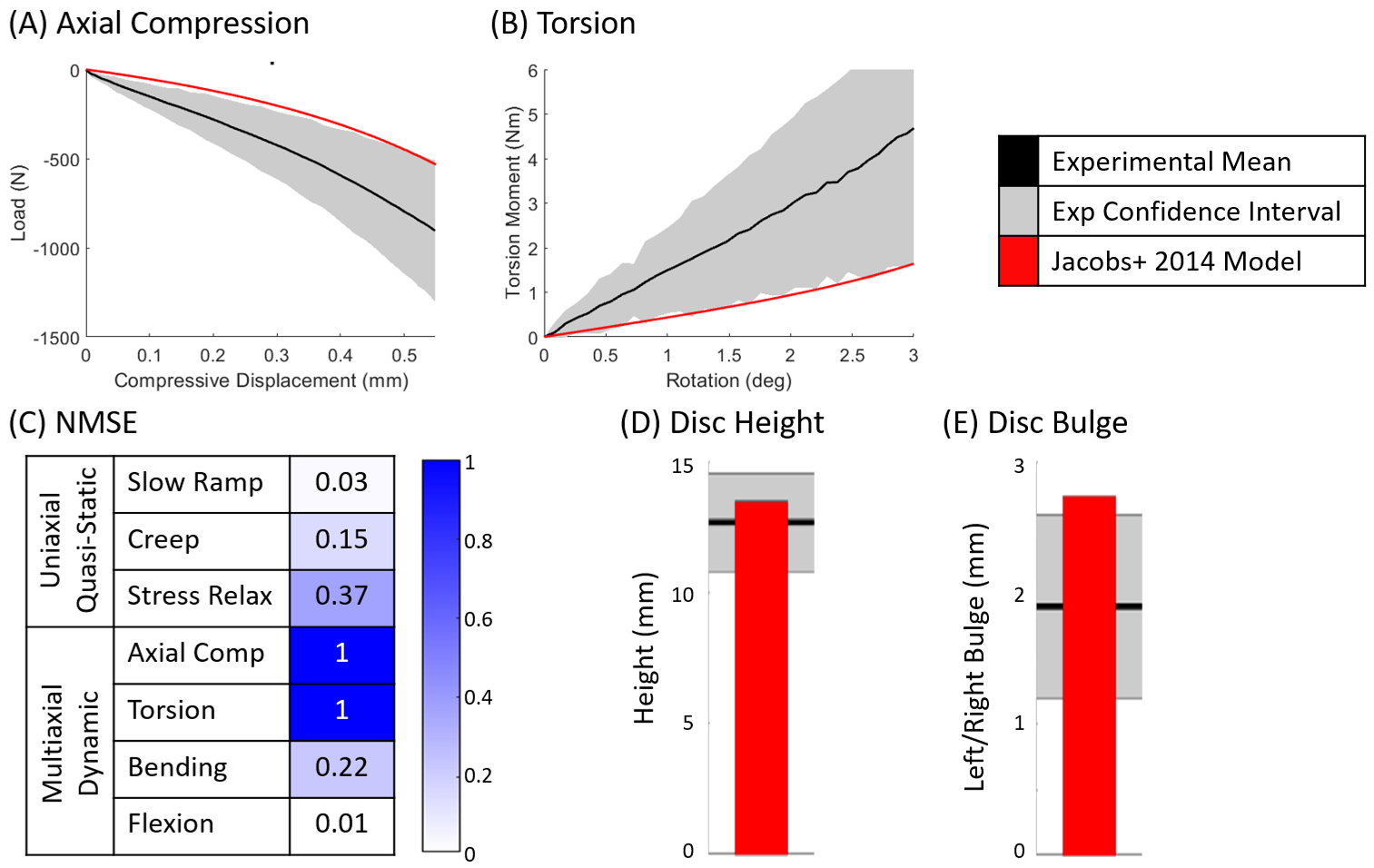

Supplement: Supplementary file 1 — Figure S1 The Jacobs+2014 model was not able to recapitulate experimental outcomes in dynamic axial compression (A) and torsion (B). C, NMSE values closer to zero indicate a model response closer to the experimental mean and NMSE value greater than 1 indicate the model response was outside the experimental 95% confidence interval. D, The model equilibrated to a 50 N load had reasonable height compared to MRI data and had mild excess bulge (E). [file JSP2-4-e1145-s002.png]

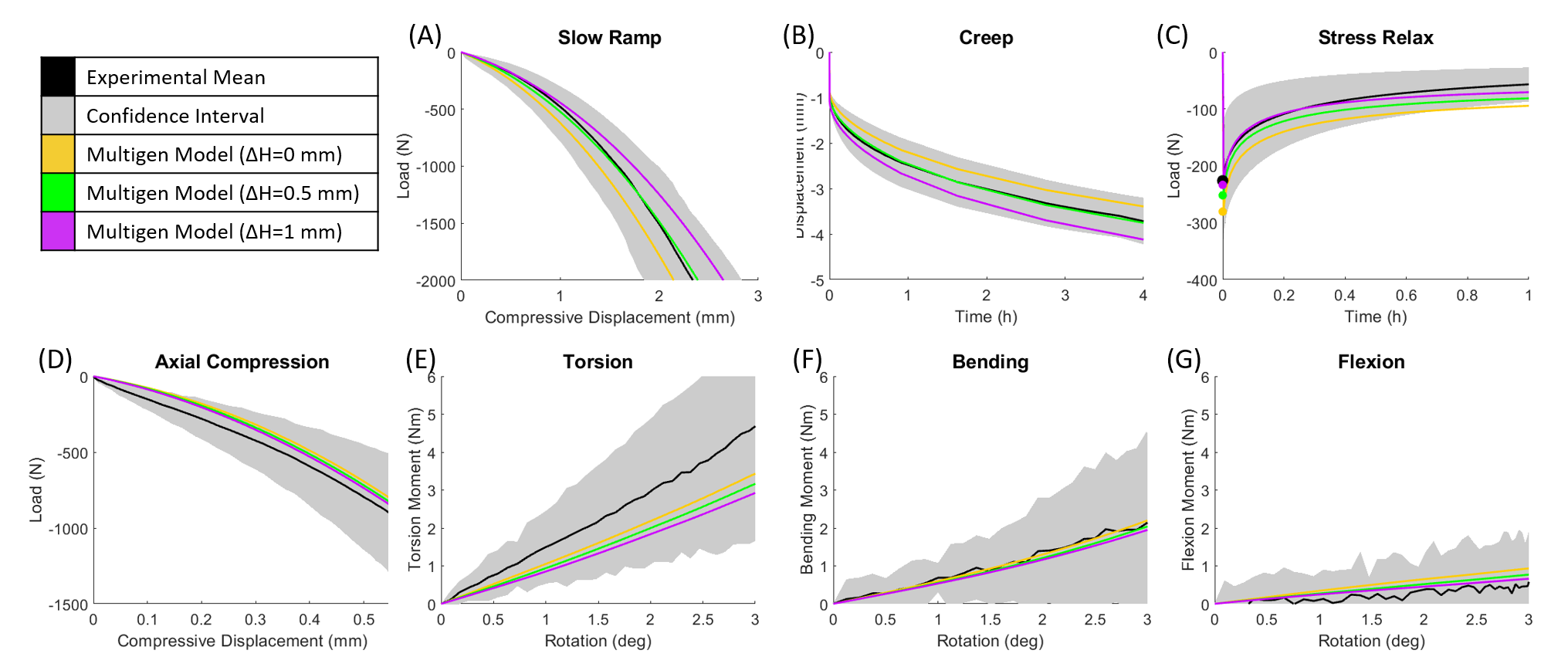

Supplement: Supplementary file 2 — Figure S2 Experimental data ±95% confidence interval is shown with model outcomes for the multigeneration models with Ω = 3° and variable axial displacement (ΔH). The model with ΔH = 0.5 mm was optimal for uniaxial quasi‐static slow ramp (A) and creep (B) outcomes. For uniaxial quasi‐static stress relaxation (C), the model with ΔH = 1 mm was best. There was minimal variation between the multigeneration model outcomes in multiaxial dynamic axial compression (D), bending (F), and flexion (G). The model with ΔH = 0 mm offered the best torsion response (E). [file JSP2-4-e1145-s004.png]

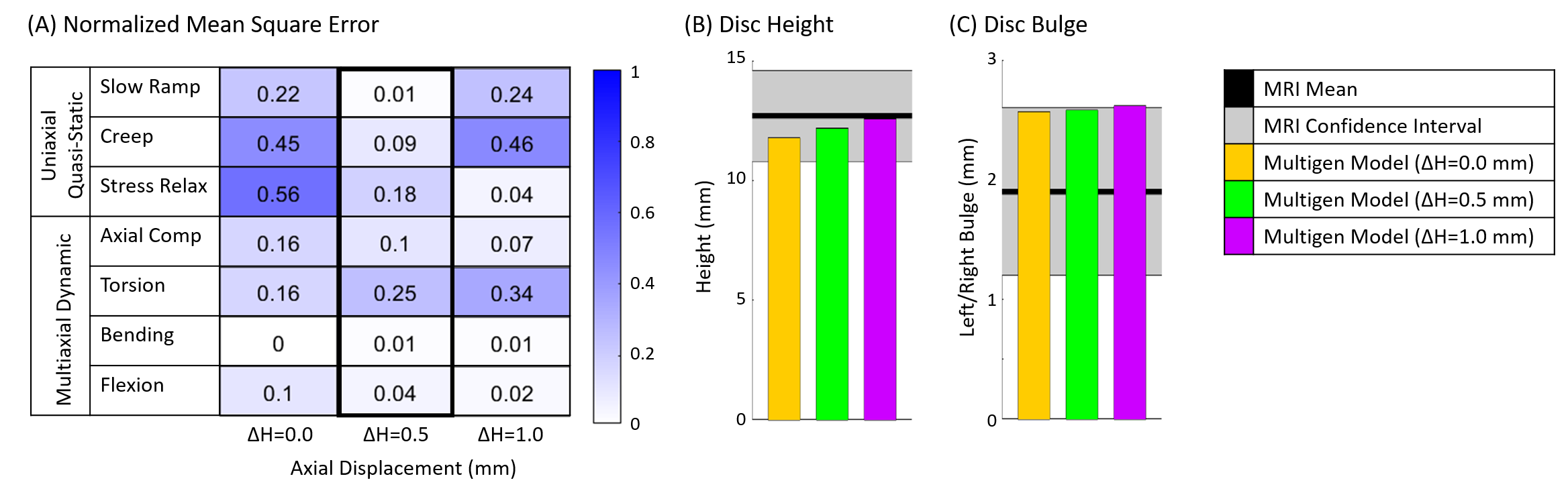

Supplement: Supplementary file 3 — Figure S3 A, Normalized mean square error (NMSE) values are shown for the multigeneration model with varying axial displacement (ΔH) with twist angle Ω = 3°. NMSE values closer to zero indicate a model response closer to the experimental mean. Axial displacement ΔH = 0.5 mm was necessary to optimize slow ramp and creep outcomes. Stress relaxation was best at ΔH = 1.0 mm while torsion was best at ΔH = 0.0 mm. All model responses were reasonable in axial compression, bending, and torsion tests. B and C, Disc height and bulge from the models equilibrated to a 50 N load compared to MRI data of discs under a 50 N load. B, All models were within expected height limits. C, All models are in the upper range of disc bulge. [file JSP2-4-e1145-s007.png]

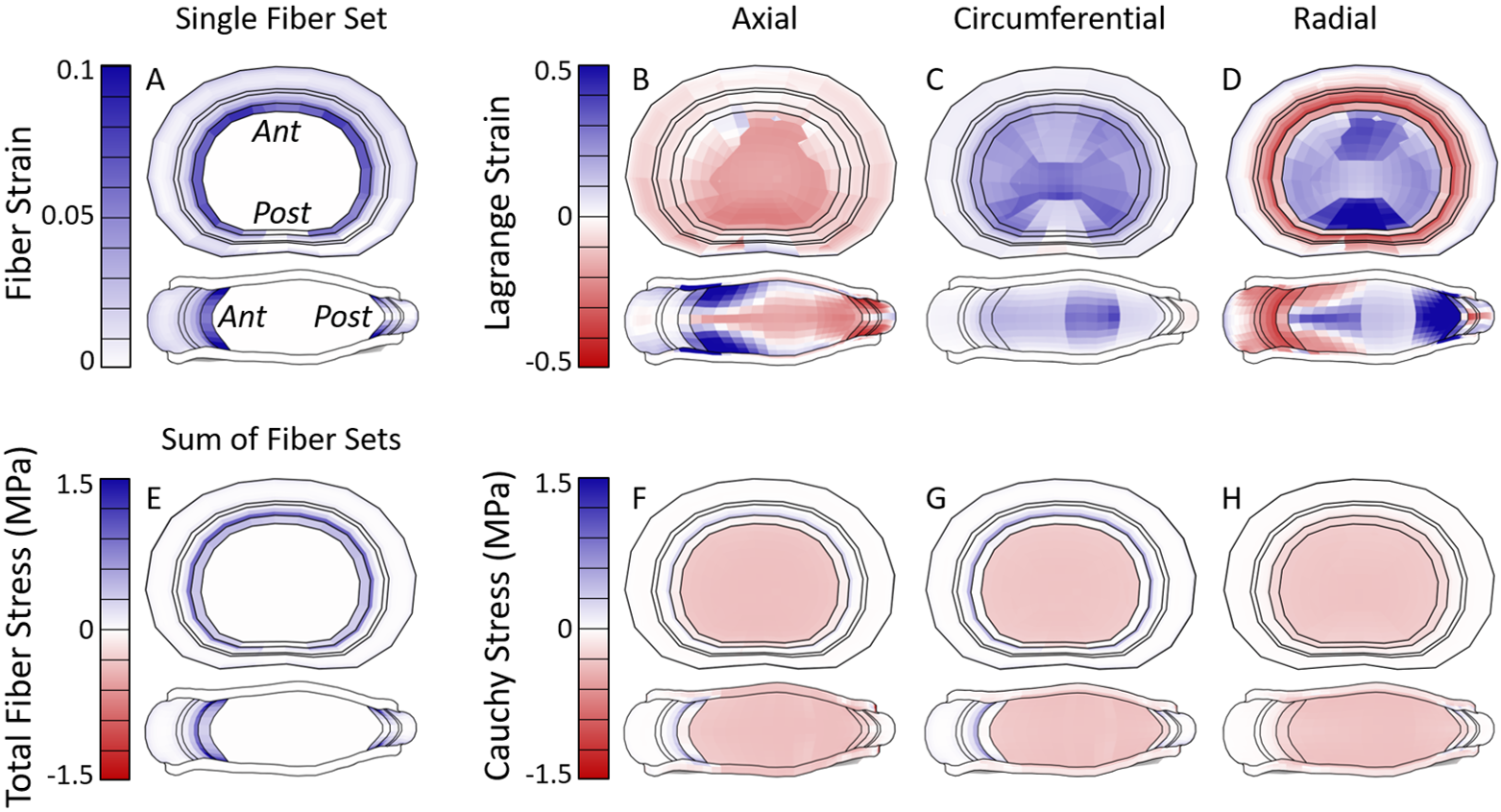

Supplement: Supplementary file 4 — Figure S4 The stress and strain state of the swelling‐only model following equilibration to a 270 N preload, a relevant pre‐test state for the multiaxial dynamic tests. The fiber strain profile was similar for both fiber sets, only one set shown. Fiber strain and Cauchy stresses (A, F‐H) are identical to those shown in Figure 7A‐D, respectively. [file JSP2-4-e1145-s005.png]

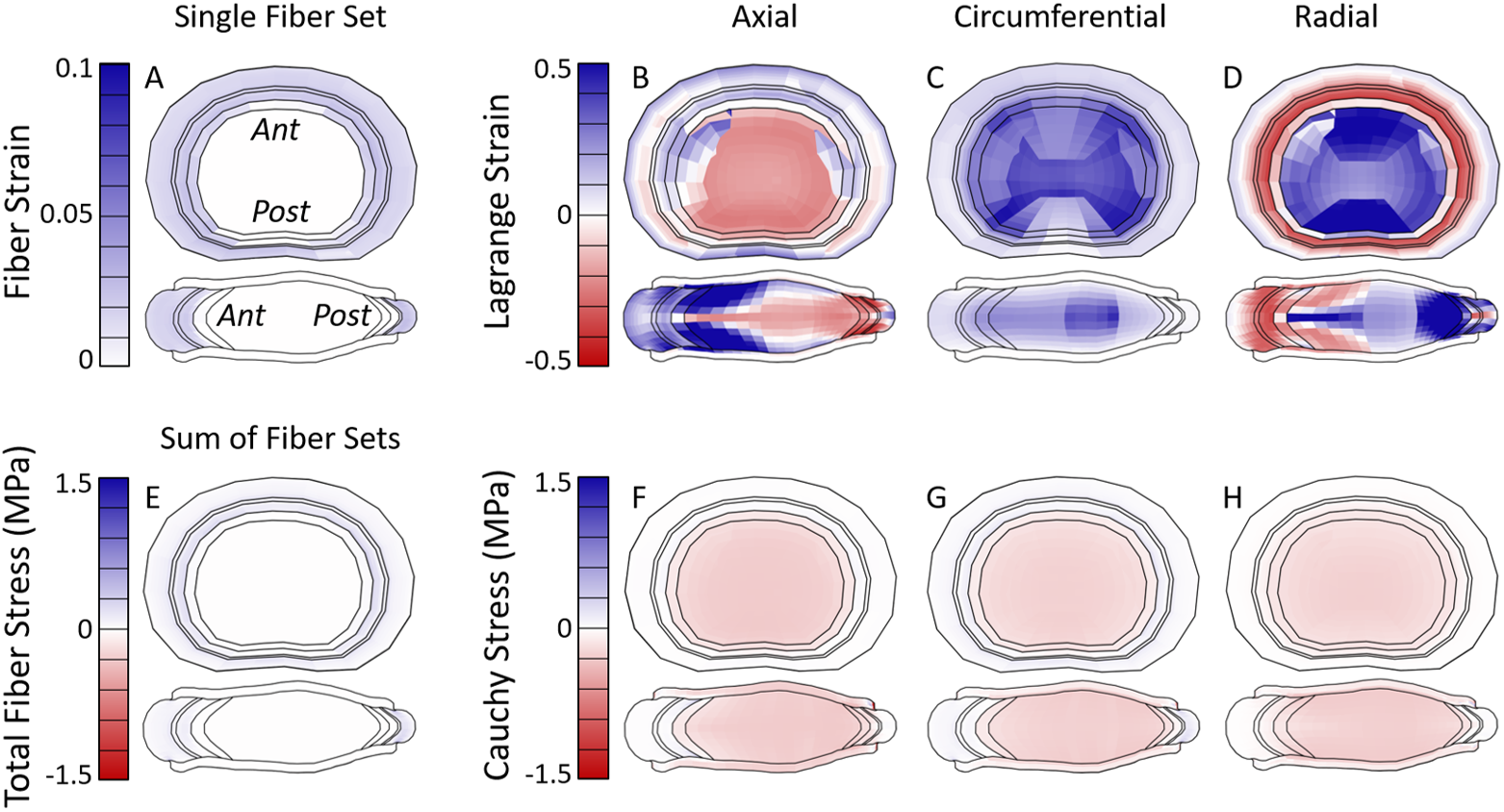

Supplement: Supplementary file 5 — Figure S5 The stress and strain state of the multigeneration model (Ω = 3°) following equilibration to a 270 N preload, a relevant pre‐test state for the multiaxial dynamic tests. The fiber strain profile was similar for both fiber sets, only one set shown. Fiber strain and Cauchy stresses (A, F‐H) are identical to those shown in Figure 7E‐H, respectively. [file JSP2-4-e1145-s011.png]

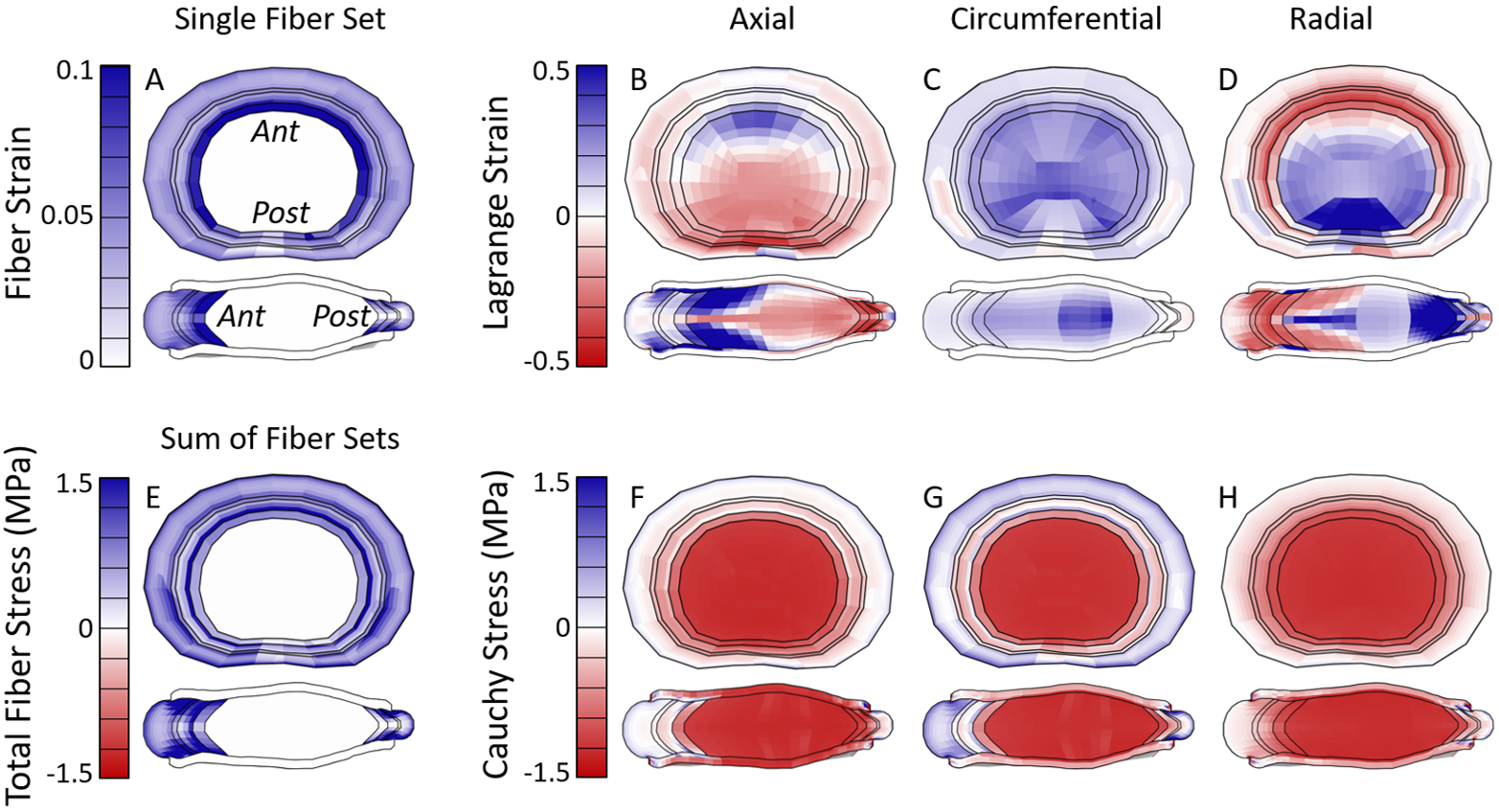

Supplement: Supplementary file 6 — Figure S6 The stress and strain state of the swelling‐only model at maximum compression from the multiaxial dynamic axial compression test. The fiber strain profile was similar for both fiber sets, only one set shown. Fiber strain and Cauchy stresses (A, F‐H) are identical to those shown in Figure 8A‐D, respectively. [file JSP2-4-e1145-s010.png]

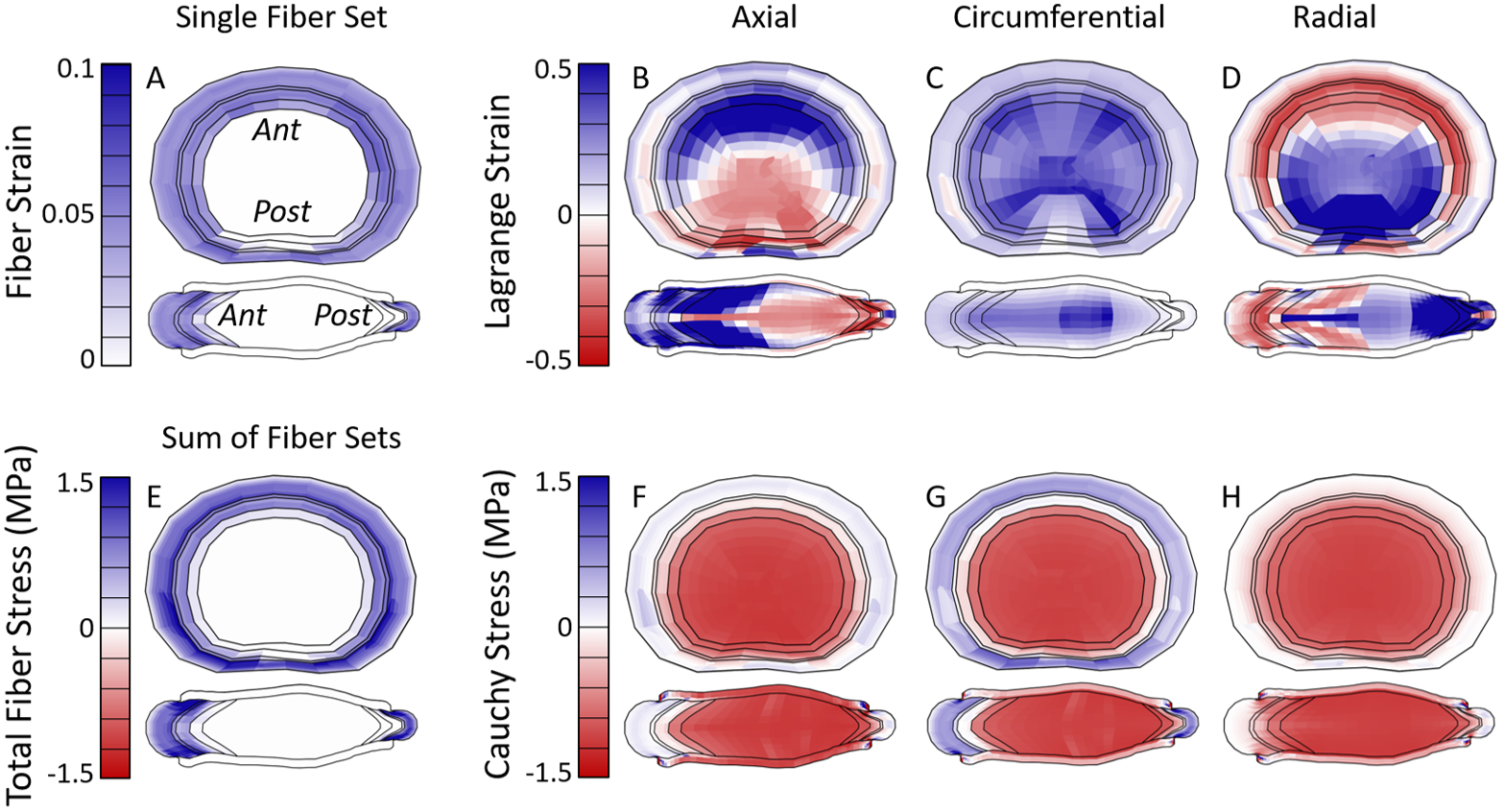

Supplement: Supplementary file 7 — Figure S7 The stress and strain state of the multigeneration model (Ω = 3°) at maximum compression from the multiaxial dynamic axial compression test. The fiber strain profile was similar for both fiber sets, only one set shown. Fiber strain and Cauchy stresses (A, F‐H) are identical to those shown in Figure 8E‐H, respectively. [file JSP2-4-e1145-s006.png]

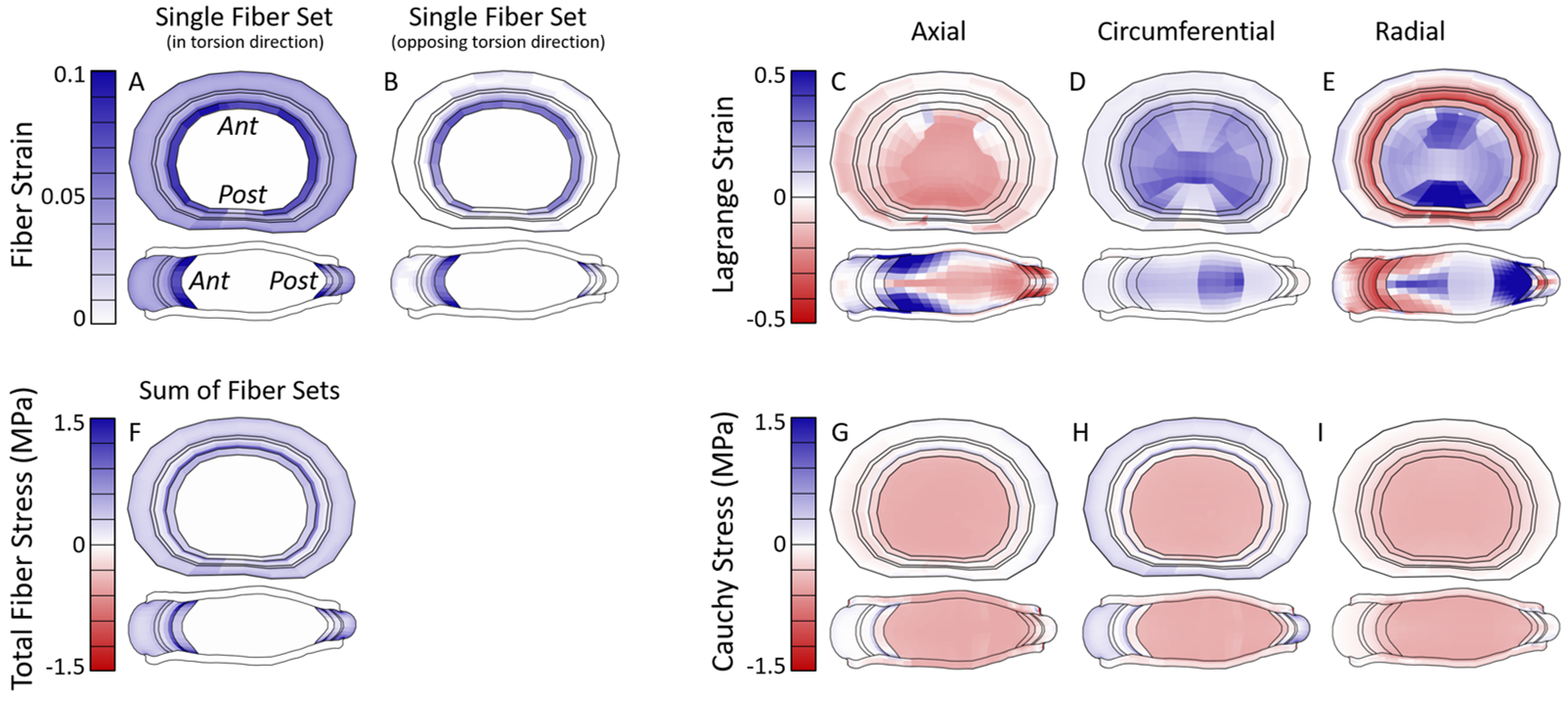

Supplement: Supplementary file 8 — Figure S8 The stress and strain state of the swelling‐only model at maximum torsion from the multiaxial dynamic torsion test. Fiber strains and Cauchy stresses (A‐B, F‐H) are identical to those shown in Figure 9A‐E, respectively. [file JSP2-4-e1145-s008.png]

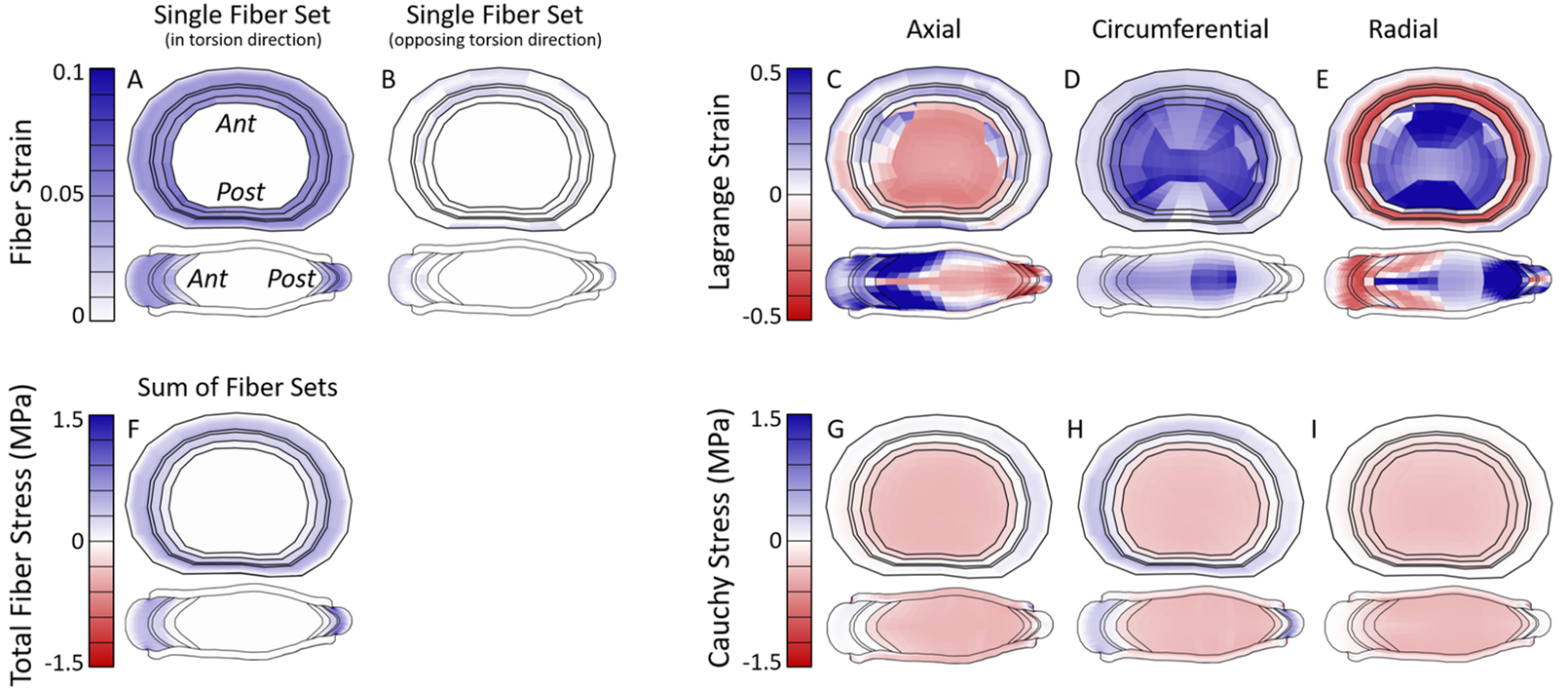

Supplement: Supplementary file 9 — Figure S9 The stress and strain state of the multigeneration model (Ω = 3°) at maximum torsion from the multiaxial dynamic torsion test. Fiber strains and Cauchy stresses (A‐B, F‐H) are identical to those shown in Figure 9F‐J, respectively. [file JSP2-4-e1145-s001.png]

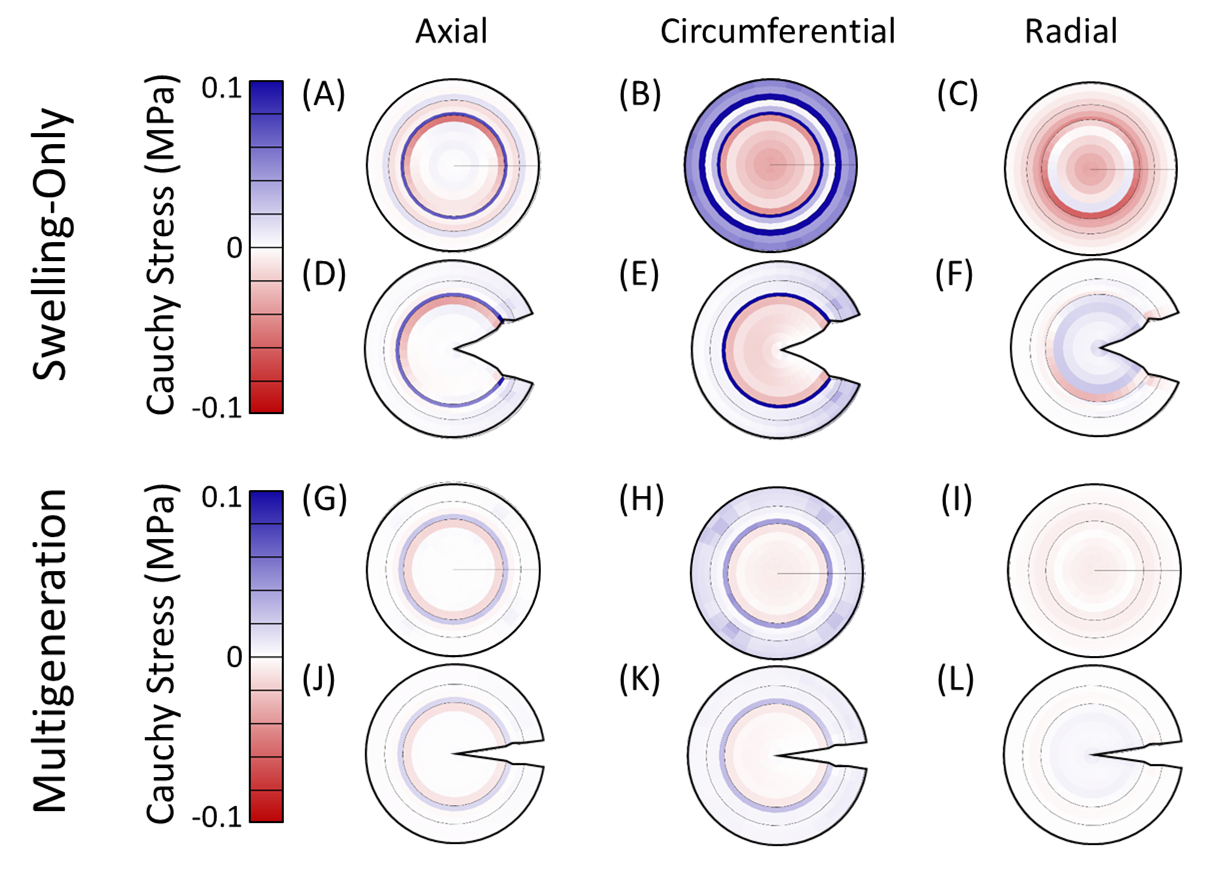

Supplement: Supplementary file 10 — Figure S10 The bovine disc model immediately before (A‐C, G‐I) and following radial incision (D‐F, J‐L) for the swelling‐only model (A‐F) and multigeneration model (G‐L). [file JSP2-4-e1145-s009.png]

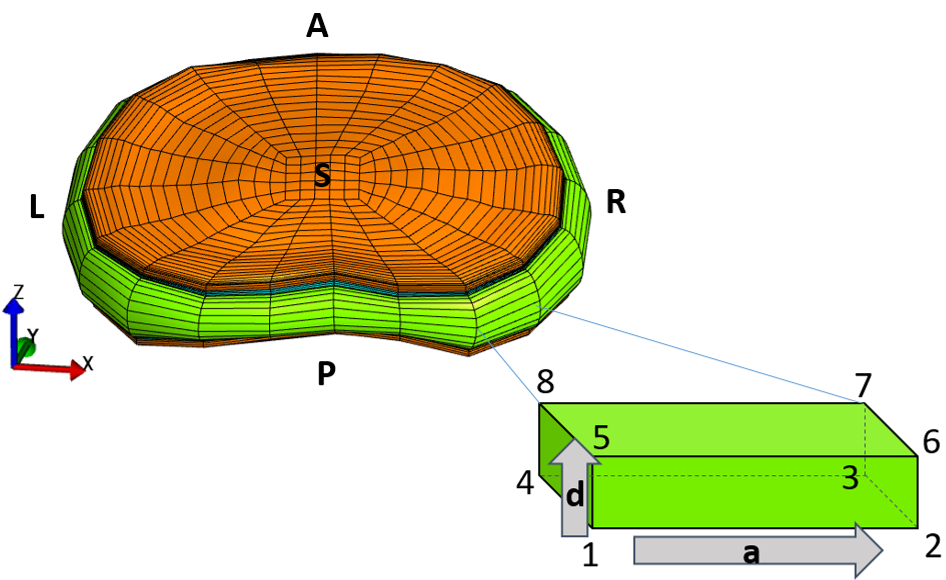

Supplement: Supplementary file 11 — Figure S11 Disc model with anterior (A), posterior (P), superior (S), left (L) and right (R) directions labeled. A schematic of a single element with local node numbers and directional vectors 'a' and 'd' shown for a local material axis definition of [1, 2, 5] for more details see FEBio user manual. [file JSP2-4-e1145-s003.png]
